# Context-dependent interactions among afadin, ZO-1, and actin filaments

**DOI:** 10.1247/csf.25019

**Published:** 2025-11-18

**Authors:** Yuji Nitta, Satoshi Urayama, Maki Kawashima, Hayato Nakao, Takafumi Ikeda, Kazushi Higashiyama, Hatsuki Murakami, Chiyoko Kobayashi, Yuichiro Kano, Mikio Furuse, Akira Nagafuchi

**Affiliations:** 1 Department of Biology, School of Medicine, Nara Medical University, 88 Shijo-cho, Kashihara, Nara 634-0813, Japan; 2 Division of Cell Structure, National Institute for Physiological Sciences, Okazaki, Aichi, Japan; 3 Physiological Sciences Program, Graduate Institute for Advanced Studies, SOKENDAI, Okazaki, Aichi, Japan

**Keywords:** afadin, ZO-1, actin, F9 cell, L cell

## Abstract

Afadin and ZO-1 are actin-binding scaffold proteins localized at cell-cell junctions. Although these proteins contain multiple protein-binding motifs for various junctional proteins, their binding partners within cells are strictly regulated. Here, we investigated the mutual interactions among afadin, ZO-1, and actin filaments using cells lacking cellular junctions derived from EL and F9 non-epithelial cells. In EL-derived cells, afadin and ZO-1 independently colocalized with various types of actin filaments. In F9-derived cells, afadin and ZO-1 colocalized as aggregates. Gene disruption analyses revealed that afadin and ZO-1 independently form aggregates in the absence of cadherin-catenin complex. Nectin-2, an afadin-binding membrane protein, was detected in afadin aggregates but not in ZO-1 aggregates, suggesting the existence of a membrane protein that binds to ZO-1. We identified this protein as JAM-C. A comparison between α-catenin-deficient and β-catenin-deficient F9 cells suggested that the extracellular domain of E-cadherin interferes with afadin and ZO-1 aggregate formation. Furthermore, gene disruption of nectin-2 suggested that JAM-C-bound ZO-1, rather than unbound ZO-1, preferentially interacts with afadin. Together, these findings indicate that interactions among afadin, ZO-1, and actin filaments are strictly regulated by various cellular contexts.

## Graphical Abstract

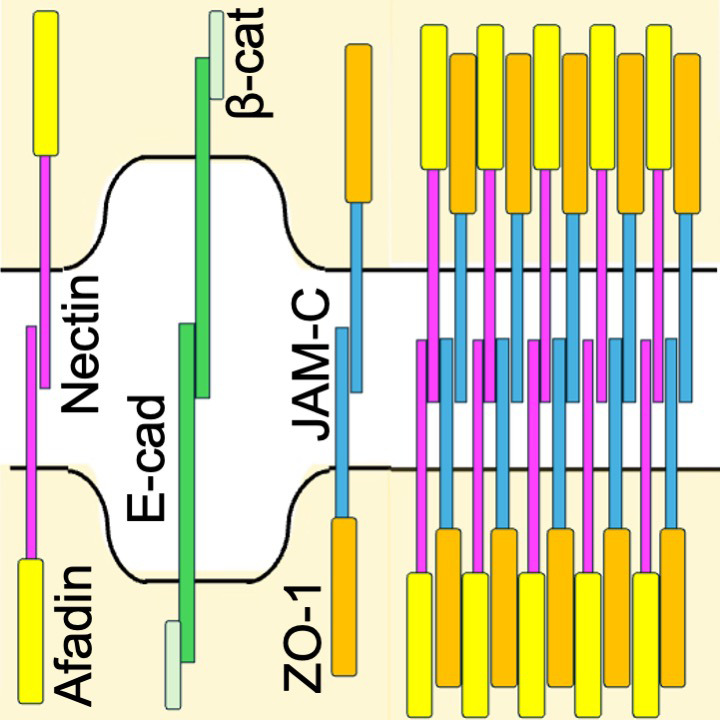

## Introduction

The cell-cell junctional complex plays a crucial role in both maintaining strong cell-cell adhesion and facilitating dynamic cell-cell rearrangement during animal morphogenesis. Initial electron microscopic analysis identified three discernable structures at adjacent polarized epithelial cells, tight junctions (TJs), adherens junctions (AJs) and desmosomes (Farquhar and Palade, 1963). Although TJs and desmosomes are primarily found in polarized epithelial cells, AJs are present in most adherent cells, including non-epithelial cells (Boda-Heggemann *et al.*, 2009; Duband *et al.*, 1987; Itoh *et al.*, 1993; Nagafuchi and Tsukita, 1994). AJs are mainly composed of cadherins, a family of cell adhesion molecules, and their cytoplasmic binding partners, β-catenin and α-catenin (Herrenknecht *et al.*, 1991; McCrea *et al.*, 1991; Nagafuchi *et al.*, 1991; Ozawa *et al.*, 1989). In addition to the cadherin-catenin complex (CCC), other components are essential for AJ formation. Notably, afadin and ZO-1 play pivotal roles in AJ formation as actin-binding scaffold proteins (Ikenouchi *et al.*, 2007; Imamura *et al.*, 1999; Itoh *et al.*, 1993; McParland *et al.*, 2024; Tachibana *et al.*, 2000; Yokoyama *et al.*, 2001)

Afadin and ZO-1 are major components of AJs and TJs, respectively, in polarized epithelial cells (Rouaud *et al.*, 2020). In non-epithelial cells, however, ZO-1 is also a major component of AJs (Imamura *et al.*, 1999; Itoh *et al.*, 1993). Although both afadin and ZO-1 contain multiple protein-binding motifs as actin-binding scaffold proteins, their binding partners are strictly regulated depending on the cellular context. In epithelial cells, for instance, ZO-1 binds to TJ components such as occludin and claudins, whereas in non-epithelial cells, it binds to α-catenin (Furuse *et al.*, 1994; Imamura *et al.*, 1999; Itoh *et al.*, 1999). On the other hand, although both afadin and ZO-1 can bind to afadin, ZO-1, and actin filaments, these proteins do not necessarily colocalized with each other (Itoh *et al.*, 1997; Liedtke *et al.*, 2010; Mandai *et al.*, 1997; Ooshio *et al.*, 2010; Utepbergenov *et al.*, 2006). Moreover, it has been extensively investigated whether phase separation is involved in the aggregate formation and localization of afadin and ZO-1 (Kuno *et al.*, 2025; Pombo-García *et al.*, 2024). To elucidate the regulatory mechanisms governing these complex interactions, it is necessary to examine their localization under various conditions.

F9 cells are a mouse teratocarcinoma cell line (Sherman and Miller, 1978). Although F9 cells can differentiate into epithelial cells under differentiation conditions, they grow as non-epithelial cells similar to ES cells under normal culture condition (Hogan *et al.*, 1981; Komiya *et al.*, 2005). To date, using classical homologous recombination, we have isolated various mutant F9 cells lacking CCC components, such as αD, which lacks α-catenin, BPD, which lacks β-catenin and plakoglobin (Fukunaga *et al.*, 2005; Maeno *et al.*, 1999). EL cells are stable transfectants of mouse fibroblast L cells expressing E-cadherin (Nagafuchi *et al.*, 1987; Nose *et al.*, 1988). L cells, identified as a cadherin-deficient cell line, have been widely used as model cells to investigate the adhesive functions of various cadherin molecules (Hatta *et al.*, 1988; Nagafuchi *et al.*, 1987, 1994; Nose *et al.*, 1988). Since the efficiency of homologous recombination is low, mutant L cells lacking α-catenin and/or β-catenin have not yet been successfully isolated. Both F9 and EL cells express afadin and ZO-1, allowing us to use these cells and their derivatives for localization analysis of these proteins (Tachibana *et al.*, 2000; Yokoyama *et al.*, 2001). Afadin and ZO-1 colocalize with CCC in F9 cells and EL cells, likely through α-catenin, as described above. To evaluate the interactions among afadin, ZO-1, and actin filaments, we must observe the localization of these proteins independently of CCC components.

In this study, we isolated EL cells lacking α-catenin (EL-αKO cells) and β-catenin (EL-βKO cells) using the CRISPR/Cas9 system. As a result, we obtained F9 and EL cell derivatives lacking α-catenin (αD and d EL-αKO) and β-catenin (BPD and EL-βKO). Since these cells lack TJs and AJs, we were able to observe the localization of afadin, ZO-1, and actin filaments in the absence of cell-cell junctions. Moreover, using these cells treated with cytochalasin D (CD) or latrunculin B (LB), we examined the effects of actin filament polymerization on the localization of afadin and ZO-1. In addition, we isolated several cell lines lacking afadin, ZO-1 and nectin-2 using the CRISPR/Cas9 system and examined the localization of afadin, ZO-1, and actin filaments. As a result, we found that various factors, including cell type, the expression of CCC components, the presence of afadin and ZO-1, so far unrecognized member of non-epithelial junction, JAM-C, and the polymerization state of actin filaments, influence the localization patterns of afadin and ZO-1.

## Materials and Methods

### Cells and Cell culture

In this study, mouse teratocarcinoma F9 cells (Sherman and Miller, 1978) and their derivatives, as well as the derivatives of mouse fibroblast L cells (Wilton R. Earle *et al.*, 1943) were used. EL cells are L cells expressing E-cadherin. In this study, ELβ1, a clone of EL cells that expresses a relatively higher amount of E-cadherin, was used as the EL cells (Nose *et al.*, 1988). F9 cells lacking α-catenin were previously reported as αD cells (Maeno *et al.*, 1999). F9 cells deficient in β-catenin and plakoglobin were previously referred to as BPD cells or 2K cells; in this study, they were referred to as BPD cells (Fukunaga *et al.*, 2005; Ozono *et al.*, 2011). F9 cells and their derivatives express a chimeric protein composed of GATA6 and the glucocorticoid receptor ligand-binding domain, which has been used in another research. Cells were cultured in Dulbecco’s modified Eagle medium (DMEM) supplemented with 10% heat-inactivated fetal calf serum. Culture dishes or coverslips for F9-derived cells were pre-coated with 0.2% gelatin for at least 10 minutes before cell seeding. For each experiment, cells were transferred to fresh culture dishes one day prior to the experiment. For CD (Fuji Film-Wako, Kyoto, Japan) and LB (Abcam, Cambridge, UK) treatments, cells were incubated with the indicated concentrations of CD or LB for 15 minutes or 1 hour before fixation. For CK666 (Selleck, Houston, TX, USA) treatment, cells were incubated with 20 or 100 μM CK666 for 30 minutes.

### CRISPR/Cas9 vector and gene disruption

Gene disruption was performed following the protocol described by Cong *et al.* (Cong *et al.*, 2013) with certain modifications. In this study, we constructed the pCAS9-PAC plasmid, which was derived from the CAG-hspCas9-H1-gRNA system (*All-in-one Cas9 SmartNuclease Plasmid*, CAS920A; System Biosciences, Palo Alto, CA, USA). In the pCAS9-PAC plasmid, the H1 promoter sequence was replaced with that of pSINsi-HH1, and a puromycin-resistance cassette was inserted between the CAG promoter and the kanamycin-resistance cassette. Target sites for the genes were selected using CRISPRdirect, with a focus on regions proximal to the translation initiation sites of the target genes. The guide sequences used are listed in Supplementary Table 1. The constructed vectors were transfected into cells using Lipofectamine 2000 (Thermo Fisher Scientific, Waltham, MA, USA). To select transiently transfected cells, puromycin selection was applied two days after transfection. Cells were initially incubated with 20–40 μM puromycin for approximately 6 hours, followed by 40–80 μM puromycin for an additional 20 hours. The puromycin concentration was adjusted according to the resistance properties of each transfected cell line. After puromycin selection, cells were cultured in the absence of puromycin until visible colonies formed. Candidate clones were first screened by immunocytochemical analysis, and gene disruption in the isolated clones was confirmed by sequence analysis. Furthermore, the expression of the target proteins in the selected clones was assessed via Western blot analysis.

### Purification of genomic DNA, genomic PCR product and sequence analysis

The genomic DNA derived from 6 cm confluent culture was isolated by NucleoSpin DNA RapidLyse (TaKaRa, Kusatsu, Japan). The genomic regions of the target genes were amplified by genomic PCR using Prime STAR GXL DNA polymerase (TaKaRa) and F and R primers listed in Supplementary Table 1. Following the removal of excess PCR primers by the use of ExoSP-IT Express PCR Product Cleanup (Thermo Fisher Scientific), PCR products were sequenced by the use of F and/or R primers, BigDye Terminator v3.1 Cycle Sequencing Kit (Thermo Fisher Scientific), and Seq Studio Genetic analyzer (Thermo Fisher Scientific).

### Antibodies

The following primary antibodies were used for immunocytochemical and Western blot analyses: rat anti-mouse E-cadherin monoclonal antibody (mAb) (ECCD-2; Shirayoshi *et al.*, 1986), rat anti-mouse α-catenin mAb (α18; Nagafuchi and Tsukita, 1994), rabbit anti-mouse α-catenin polyclonal antibody (pAb) (C2081; Sigma-Aldrich, St. Louis, MO, USA), mouse anti-rat ZO-1 mAb (T8-754; Itoh *et al.*, 1993), rabbit anti-ZO-2 pAb (71-1400; Thermo Fisher Scientific) mouse anti-β-catenin mAb (610154; BD Transduction Laboratories, Franklin Lake, NJ, USA), rabbit anti-mouse l/s-Afadin pAb (A0224; Sigma-Aldrich) and rat anti-nectin-2 mAb (502-57; Santa Cruz Biotechnology, Inc., Dallas, TX, USA). Rabit anti-mouse JAM-C pAbs were generated as follows. A synthetic peptide corresponding to the C-terminal 20 amino acids of JAM-C, with an additional N-terminal cysteine (CVNYIRTSEEGDFRHKSSFVI), was conjugated to keyhole limpet hemocyanin (Eurofins Genomics Inc., Tokyo, Japan) and used as antigen. Immunization of rabbits with the peptide and serum collection were performed by Kiwa Laboratory Animal Co., LTD. (Kaiso-gun, Japan). JAM-C-specific immunoglobulins were affinity-purified from the serum using the same peptide conjugated to Sepharose beads (Thiopropyl Sepharose^TM^6B Lab Pack, Cytiva, Tokyo, Japan). For secondary antibodies, CF488- or CF568-conjugated donkey anti-mouse, anti-rabbit, and anti-rat IgG (Biotium, San Francisco, CA, USA) were used.

### Western blot analysis

SDS-PAGE and immunoblotting were performed as previously described (Imamura *et al.*, 1999). Cultured cells (1-day-old) were washed with HEPES-buffered Ca^2+^, Mg^2+^-free saline (HCMF; Takeichi, 1977) and immediately solubilized and boiled in SDS sample buffer (0.125 M Tris-HCl, pH 6.8, 2% SDS, 10% glycerol, 0.002% bromophenol blue, and 5% 2-mercaptoethanol). Proteins lysates from about 6 × 10^4^ cultured cells were separated by SDS-PAGE and transferred onto nitrocellulose membranes via electrophoresis. The membranes were then sequentially incubated with primary and secondary antibodies. Secondary antibody detection was performed using a biotin-streptavidin kit (GE Healthcare, Chicago, IL, USA) according to the manufacturer’s instructions. Representative results are shown for each experiment.

### Immunocytochemical analysis

1–4× 10^5^ cells, cultured for one day on 15-mm coverslips, were fixed, permeabilized, and then sequentially incubated with primary and secondary antibodies as previously described (Komiya *et al.*, 2005). For F9-derived cells, coverslips were coated with 0.2% gelatin solution. Cells were fixed with 2% (for anti-JAM-C pAb) or 3.3% formaldehyde solution for 15 min and permeabilized with 0.1% Triton X-100 for 10 minutes. To stain actin filaments, samples on coverslips were incubated with CF568-labeled phalloidin (Biotium) instead of the secondary antibody. Samples were mounted onto slides using Fluoro-KEEPER Antifade Reagent, Non-Hardening Type (Nacalai Tesque, Kyoto, Japan). Fluorescence images were acquired using an Axiovert 200 inverted microscope (Carl Zeiss, Oberkochen, Germany) equipped with either a 63×/1.40 oil objective or a 40×/0.75 objective. Brightness, contrast, and final image size were adjusted using GNU Image Manipulation Program. Representative images are shown for each cell line.

### Image Analysis (see Supplementary Materials and Methods for details)

Binarization of images was performed primarily using the Yen or Otsu method for EL-derived cells, and MaxEntropy method for F9-derived cells. Binarized images from different channels were compared on a pixel-by-pixel basis for colocalization analysis, and statistical significance was evaluated using the Mann-Whitney U test. For quantification of actin particle numbers, the actin channel was preprocessed with contrast-limited adaptive histogram equalization and a Gaussian filter, followed by generation of cell area masks using the Otsu method. Aggregate density values were obtained on a per-image basis, and these image-level values were used for statistical comparison across groups. For geometric analysis, actin particles were used as the reference, and afadin-actin or ZO-1-actin pairs were evaluated as target combinations. Distance was defined as the minimum distance between boundary pixels of the reference and target particles. Image processing and quantitative analyses were performed using Fiji (ImageJ; version 1.54p, National Institutes of Health, Bethesda, MD, USA) and Python (version 3.9.6) with the SciPy and pandas libraries.

## Results

### Isolation of EL- and F9-derived cell lines lacking α-, β-catenin, afadin, ZO-1 and/or nectin-2

**Fig. 1:**
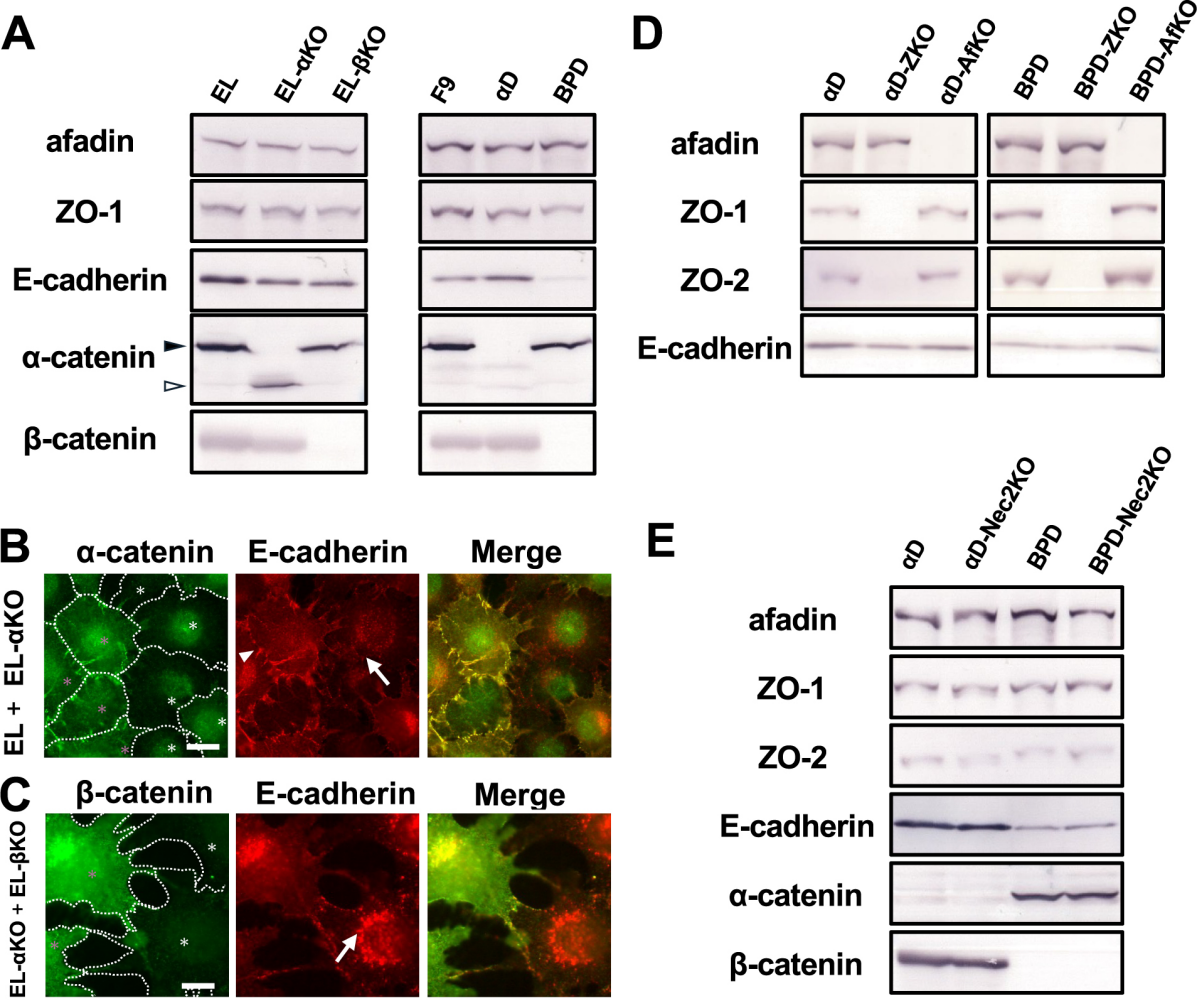
EL- and F9-derived cells lacking α-, β-catenin, afadin, ZO-1, and/or nectin-2 (A) Western blot analysis using antibodies against afadin, ZO-1, E-cadherin, α-catenin, and β-catenin in EL cells, EL-derived cells, F9 cells, and F9-derived cells. The black arrowhead indicates full-length α-catenin, while the white arrowhead indicates a truncated form observed in EL-αKO cells. (B) Immunostaining of co-cultured EL and EL-αKO cells. Green: α-catenin, red: E-cadherin. Magenta asterisks indicate EL cells, and white asterisks indicate EL-αKO cells. The arrowhead indicates cell-cell contact sites between EL cells, while the arrow indicates those between EL-αKO cells. Scale bar = 20 μm. (C) Immunostaining of co-cultured EL-αKO and EL-βKO cells. Green: β-catenin, red: E-cadherin. Magenta asterisks indicate EL-αKO cells, and white asterisks indicate EL-βKO cells. The arrow indicates cytoplasmic E-cadherin aggregates in EL-βKO cells. Scale bar = 20 μm. (D) Western blot analysis using antibodies against afadin, ZO-1, ZO-2, and E-cadherin in αD cells, αD-derived cells, BPD cells, and BPD-derived cells. (E) Western blot analysis using antibodies against afadin, ZO-1, ZO-2, E-cadherin, α-catenin, and β-catenin in αD cells, αD-Nec2KO cells, BPD cells, and BPD-Nec2KO cells.

In this study, as shown below, we generated several gene-disrupted cell lines using the CRISPR/Cas9 system. To achieve this, we constructed a pCAS9-PAC plasmid and selected target sequences for each gene using CRISPRdirect (see Materials and Methods; Supplementary Table 1). The pCAS9-PAC vector containing the appropriate target sequence was transfected into EL cells and two F9-derived cell lines, αD cells and BPD cells. Two days after transfection, the cells were transiently treated with puromycin. After colony formation, we screened cell lines lacking expected protein expression using immunohistochemical analysis. The loss of protein expression was further confirmed by immunoblot analysis (Fig. 1A and D) and the detection of in-del mutations in the target gene by sequencing genomic PCR product amplified using specific forward and reverse primers (Supplementary Table 1). Finally, we selected three to five clones for each gene. Using this strategy, we newly isolated and characterized 8 lines as described below. Together with αD, BPD, and parental F9, and EL cells, we prepared 12 cell lines used in this study, as summarized in Table 1.

To analyze the localization of afadin, ZO-1 and actin filaments in the absence of CCC components, we newly established two EL-derived cell lines, α-catenin-deficient EL cells (EL-αKO cells) and β-catenin-deficient EL cells (EL-βKO cells). The truncated form of α-catenin observed in EL-αKO cells likely lacks the N-terminal beta-catenin-binding domain (Fig. 1A). Previously, we isolated F9-derived α-catenin- and β-catenin-deficient cells (αD and BPD cells, respectively). By comparing EL-derived cells to F9-derived cells, we identified two key differences in the role of α-catenin and β-catenin in E-cadherin expression and localization. In F9-derived cells, it has been reported that β-catenin, but not α-catenin, is required for the localization of E-cadherin at cell-cell contact sites. In this context, E-cadherin is detected at cell-cell contact sites in αD cells, but not in BPD cells. However, in EL-derived cells, α-catenin is necessary for the condensed localization of E-cadherin at cell-cell contact sites. In EL-αKO cells, E-cadherin fails to exhibit clear localization at cell-cell contact sites (Fig. 1B). Despite this, the expression levels of E-cadherin and β-catenin in EL-αKO cells were comparable to those in EL cells (Fig. 1A), suggesting that α-catenin is required for the condensed localization of CCC at cell-cell contact sites, but not for the expression of E-cadherin and β-catenin proteins. Indeed, in EL-αKO cells, the E-cadherin formed small aggregates in the cytoplasm and exhibited weak condensation at cell-cell contact planes (Fig. 1B and C). On the other hand, it has been reported that β-catenin is essential for the stable expression of E-cadherin in F9-derived cells. In BPD cells, the expression level of E-cadherin is markedly reduced (Fig. 1A). However, in EL-derived cells, the loss of β-catenin expression does not affect the overall expression level of E-cadherin. In EL-βKO cells, E-cadherin was observed in the cytoplasm as granule-like structures (Fig. 1C).

To analyze the localization of afadin and ZO-1 in the absence of ZO-1 or afadin, respectively, we established four F9-derived cells, afadin-deficient αD and BPD cells (αD-AfKO and BPD-AfKO cells, respectively), ZO-1/ZO-2-deficient αD and BPD cells (αD-ZKO and BPD-ZKO cells, respectively). In these cells, the loss of target proteins did not affect E-cadherin, afadin and/or ZO-1 expression levels (Fig. 1D).

To analyze the localization of afadin and ZO-1 in the absence of nectin-2, we established two F9-derived cells, nectin-2-deficient αD (αD-Nec2KO) and nectin-2-deficient BPD cells (BPD-Nec2KO). Since the anti-nectin-2 antibody is not functional for our western blot analysis, gene disruption in the isolated clones was confirmed only by sequence analysis. In these cells, the loss of target proteins did not affect E-cadherin, afadin, ZO-1, ZO-2, α-catenin and/or β-catenin expression levels (Fig. 1E).

### Localization of afadin, ZO-1, and actin filaments in EL-αKO and EL-βKO cells

**Fig. 2:**
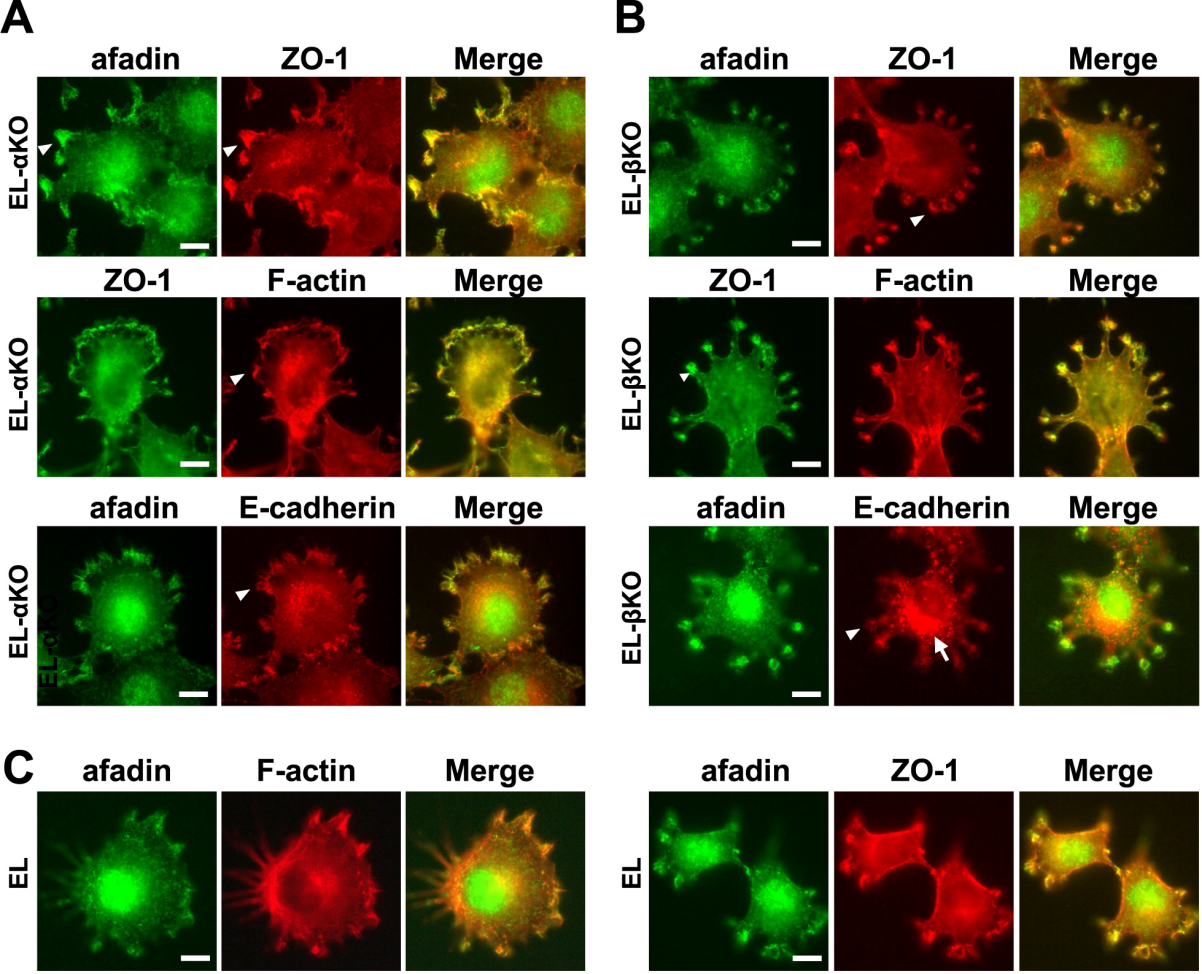
Localization of afadin, ZO-1, and actin filaments in EL, EL-αKO and EL-βKO cells (A)Immunostaining of EL-αKO cells. Upper panels: green, afadin; red, ZO-1, Middle panels: green, ZO-1; red, actin filaments (F-actin). Lower panels: green, afadin; red, E-cadherin. Arrowheads indicate lamellipodia-like structures. Scale bar = 10 μm. (B) Immunostaining of EL-βKO cells. Upper panels: green, afadin; red, ZO-1. Middle panels: green, ZO-1; red: actin filaments (F-actin). Lower panels: green, afadin; red, E-cadherin. Arrowheads indicate lamellipodia-like structures, and the arrow indicates cytoplasmic granules. Scale bar = 10 μm. (C) Immunostaining of EL cells cultured in low-density. Left panels: green, afadin; red, actin filaments (F-actin). Right panels: green, afadin; red, ZO-1. Scale bar = 10 μm.

First, we examined the localization of afadin, ZO-1 and actin filament in EL-αKO, EL-βKO cells, and parental EL cells (Fig. 2). In EL-αKO cells, which lack condensed junctional E-cadherin-β-catenin complexes at cell-cell contact sites, afadin showed weak condensation at lamellipodia-like structures (Fig. 2A). ZO-1 exhibited a ubiquitous distribution in both the nucleus and cytoplasm, including lamellipodia-like structures. Actin filaments were also detected throughout the cytoplasm, including these structures. The E-cadherin was weakly condensed at lamellipodia-like structures, in addition to forming cytoplasmic granules. In EL-βKO cells, E-cadherin was mainly localized in cytoplasmic granules and was absent from lamellipodia-like structures (Fig. 2B). However, similar to EL-αKO cells, afadin, ZO-1, and actin filaments were detected at lamellipodia-like structures (Fig. 2B). In addition, the endogenous α-catenin signal in EL-βKO cells was barely detectable, similar to that in EL-αKO cells (Supplementary Fig. 1). Even in parental EL cells, lamellipodia-like structures were observed when the cells were cultured in one-tenth the usual density and single, isolated cells were examined. In these structures, afadin, ZO-1, and actin filaments were detected (Fig. 1C). These findings suggest that, both in the presence and absence of CCC components, afadin colocalizes with ZO-1 and actin filaments at lamellipodia-like structures, while α-catenin does not localize to these structures in either EL cells or EL-derived cells. Furthermore, these results indicate that CCC components are not required for afadin localization at lamellipodia-like structures.

### Localization of afadin, ZO-1, and actin filaments in cells treated with CD and LB

In EL-derived cells, afadin co-localized with actin filaments only at lamellipodia-like structures. Although afadin is an actin-binding protein, it did not colocalize with most of the actin filaments present in the cytoplasm. This observation implies that afadin interacts only with actin filaments that are in a certain structure state, such as being properly polymerized or post-translationally modified. To test this possibility, we treated EL-βKO cells with different concentrations of CD or LB, both inhibitors of actin polymerization, and then observed the localization of afadin, ZO-1 and actin filaments. In CD- or LB-treated cells, actin filaments exhibited different distribution patterns depending on the concentration of the inhibitors. However, the expression levels of afadin and ZO-1 remained unchanged, even at the highest concentrations tested (Supplementary Fig. 2A–C).

**Fig. 3:**
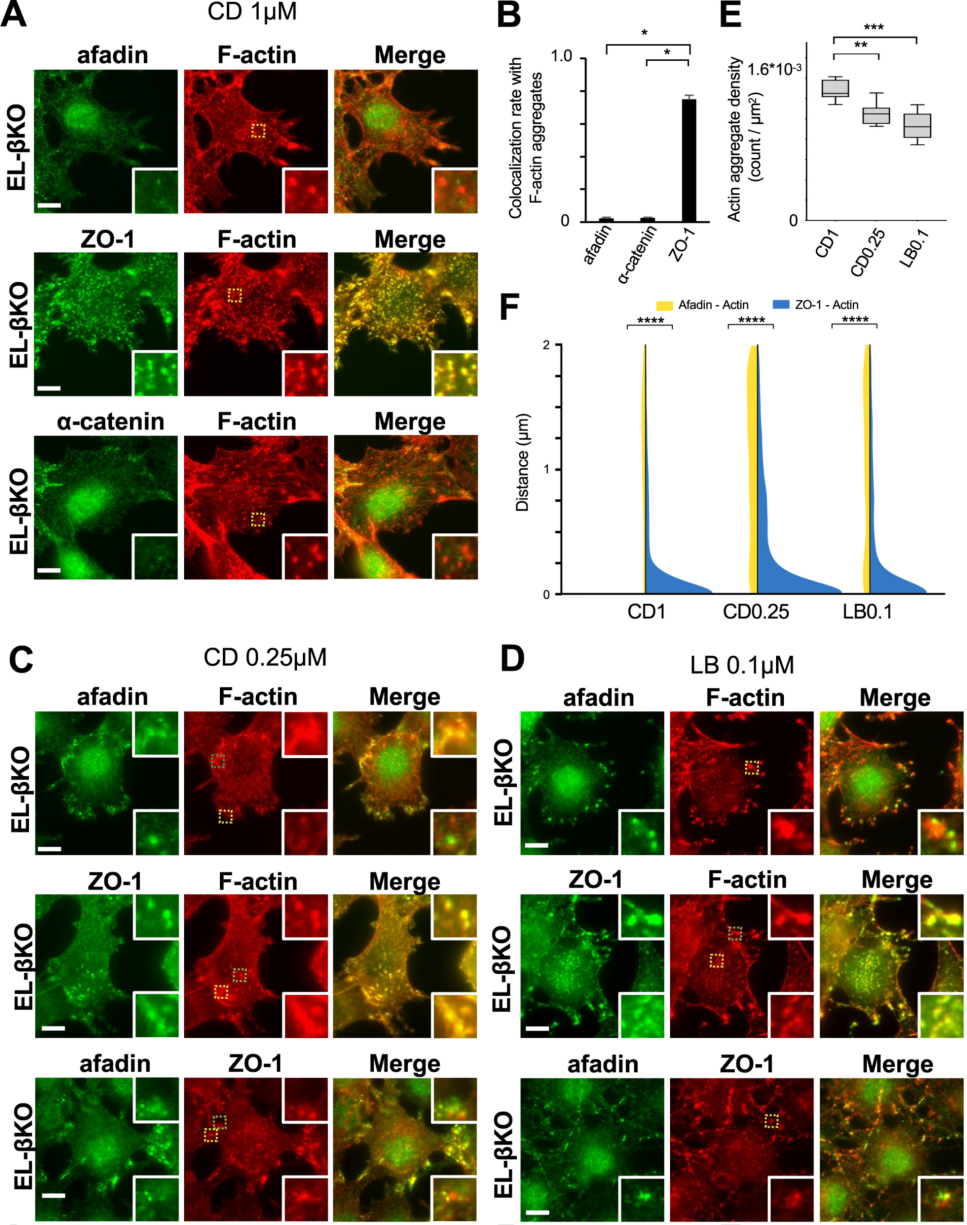
Localization of afadin, ZO-1, and actin filaments in EL-βKO cells treated with CD and LB (A) Immunostaining of EL-βKO cells treated with 1 μM CD for 15 minutes. Upper panels: green, afadin; red, actin filaments (F-actin). Middle panels: green, ZO-1; red, actin filaments (F-actin). Lower panels: green, α-catenin; red, actin filaments (F-actin). Enlarged views of the yellow-marked regions are shown in the bottom right. Scale bar = 10 μm. (B) Quantification of colocalization rates of afadin, α-catenin and ZO-1 with actin filament aggregates. ZO-1 showed significantly higher colocalization (*p<0.05, Mann-Whitney U test). Error bars represent SD. (C) Immunostaining of EL-βKO cells treated with 0.25 μM CD for 15 minutes. Upper panels: green, afadin; red, actin filaments (F-actin). Middle panels: green, ZO-1; red, actin filaments (F-actin), Lower panels: green, afadin; red, ZO-1. Enlarged views of the green-marked regions are shown in the top right, and those of the yellow-marked regions in the bottom right. Scale bar = 10 μm. (D) Immunostaining of EL-βKO cells treated with 0.1 μM LB for 1 hour. Upper panels: green, afadin; red, actin filaments (F-actin). Middle panels: green, ZO-1; red, actin filaments (F-actin). Lower panels: green, afadin; red, ZO-1. Enlarged views of the green-marked regions are shown in the top right, and those of the yellow-marked regions in the bottom right. Scale bar = 10 μm. In certain enlarged views shown in (C) and (D), afadin aggregates were observed adjacent to ZO-1/actin filament aggregates. (E) Quantification of actin aggregate density in EL-βKO cells treated with 1 μM and 0.25 μM CD or 0.1 μM LB (CD1, CD0.25, and LB0.1, respectively). A significantly higher aggregate density was observed in EL-βKO cells treated with 1 μM CD. (**p<0.01, ***p<0.001, Mann-Whitney U test). (F) Distance of afadin-actin and ZO-1-actin pairs in EL-βKO cells treated with 1 μM and 0.25 μM CD or 0.1 μM LB (CD1, CD0.25, and LB0.1, respectively). ZO-1-actin pairs exhibited significantly lower distance compared to afadin-actin pairs (****p<0.0001, Mann-Whitney U test).

When EL-βKO cells were treated with a high concentration of CD (1 μM), relatively large number of actin filament aggregates was formed (Fig. 3A, E and Supplementary Fig. 2B). In these cells, afadin hardly colocalized with the actin filament aggregates. In contrast, ZO-1 exhibited almost complete colocalization with these aggregates. Additionally, α-catenin signals were barely detectable in the actin filament aggregates (Fig. 3A and B). When EL-βKO cells were treated with a medium concentration of CD (0.25 μM), relatively smaller number of actin filament aggregates was formed compared to those in high-concentration CD-treated cells (Fig. 3C, E and Supplementary Fig. 2B). In these cells, ZO-1 aggregates were colocalized with actin filament aggregates. In this condition, afadin also frequently formed aggregates (Fig. 3C). However, these afadin aggregates did not necessarily colocalize with ZO-1/actin filament aggregates. Instead, they were often observed adjacent to ZO-1/actin filament aggregates (Fig. 3C and F). Double staining with anti-afadin and anti-ZO-1 antibodies frequently revealed that the aggregates of these proteins were located next to each other (Fig. 3C and F). These results indicate that afadin and ZO-1 have different affinities for the actin filament aggregates induced by CD treatment. Furthermore, the findings suggest that endogenous α-catenin does not interact with actin filaments, afadin and ZO-1 in these aggregates.

**Fig. 4:**
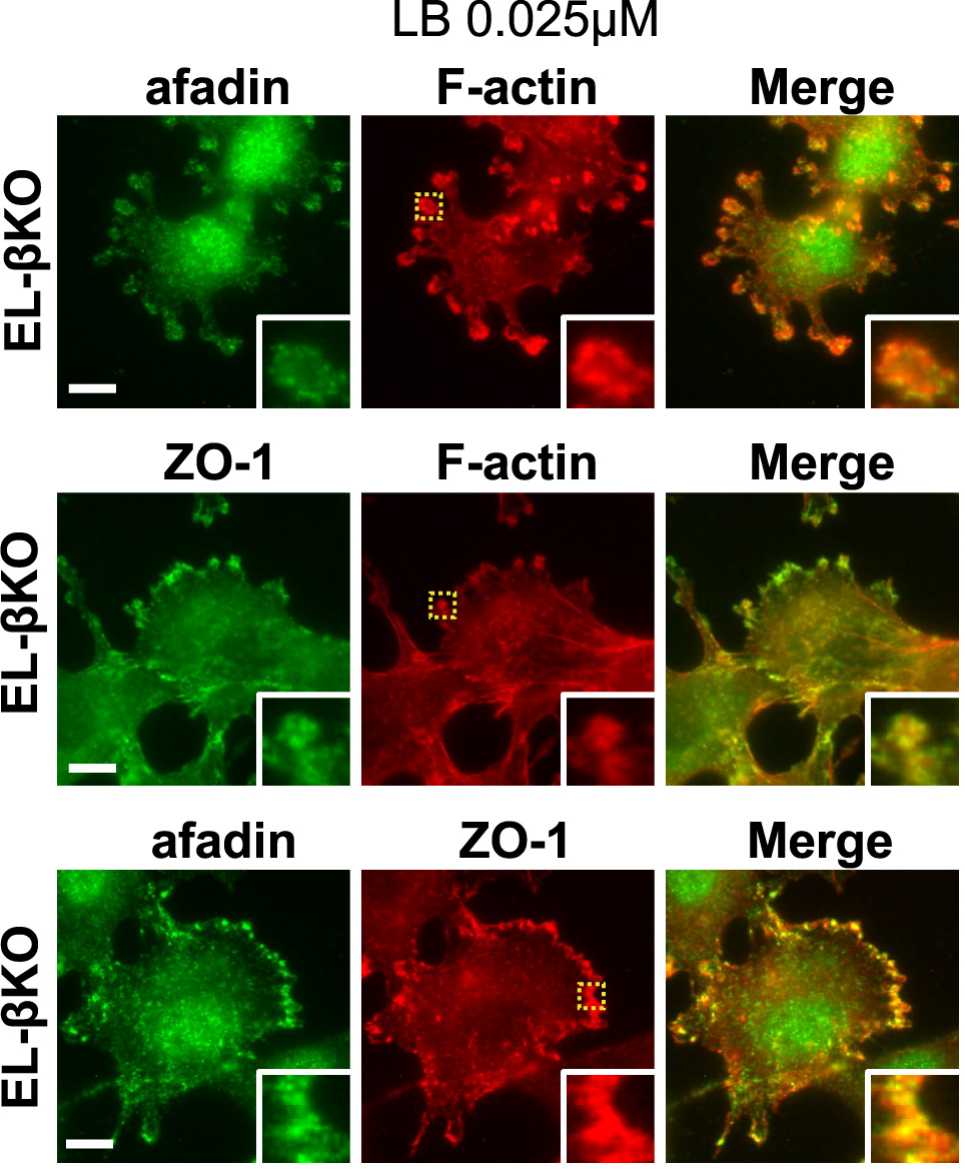
Localization of afadin, ZO-1, and actin filaments in EL-βKO cells treated with 0.025 μM LB Immunostaining of EL-βKO cells treated with 0.025 μM LB for 1 hour. Upper panels: green, afadin; red, actin filaments (F-actin). Middle panels: green, ZO-1; red, actin filaments (F-actin). Lower panels: green, afadin; red, ZO-1. Enlarged views of the yellow-marked regions are shown in the bottom right. Scale bar = 10 μm.

Next, we treated EL-βKO cells with a medium concentration (0.1 μM) and low concentration (0.025 μM) of LB and observed the localization of afadin, ZO-1, and actin filaments. When EL-βKO cells were treated with medium concentration of LB, actin filament aggregates were frequently observed (Fig. 3D and Supplementary Fig. 2C). Similar to EL-βKO cells treated with a low concentration of CD, ZO-1 aggregates were formed and colocalized with actin filament aggregates. On the other hand, afadin aggregates were formed but did not necessarily colocalize with ZO-1/actin filament aggregates and were often observed adjacent to ZO-1/actin filament aggregates (Fig. 3D and F). Moreover, they mainly observed at cell peripheries (Fig. 3D). Double staining with anti-afadin and anti-ZO-1 antibodies frequently revealed that the aggregates of these proteins were located adjacent to each other (Fig. 3D and F). When EL-βKO cells were treated with a low concentration of LB, we observed a completely different distribution of afadin, ZO-1, and actin filaments (Fig. 4). In this case, actin filament aggregates were not prominent. Instead, relatively stronger actin filament signals were observed at lamellipodia-like structures (Fig. 4 and Supplementary Fig. 2C). At these structures, both afadin and ZO-1 signals were detected. In some cells lacking lamellipodia-like structures, condensed localization of afadin and ZO-1 was observed at cell periphery (Fig. 4). These results suggest that afadin and ZO-1 have different affinities for different states of actin filament polymerization.

### Localization of afadin, ZO-1, and actin filaments in cells treated with CK666

**Fig. 5:**
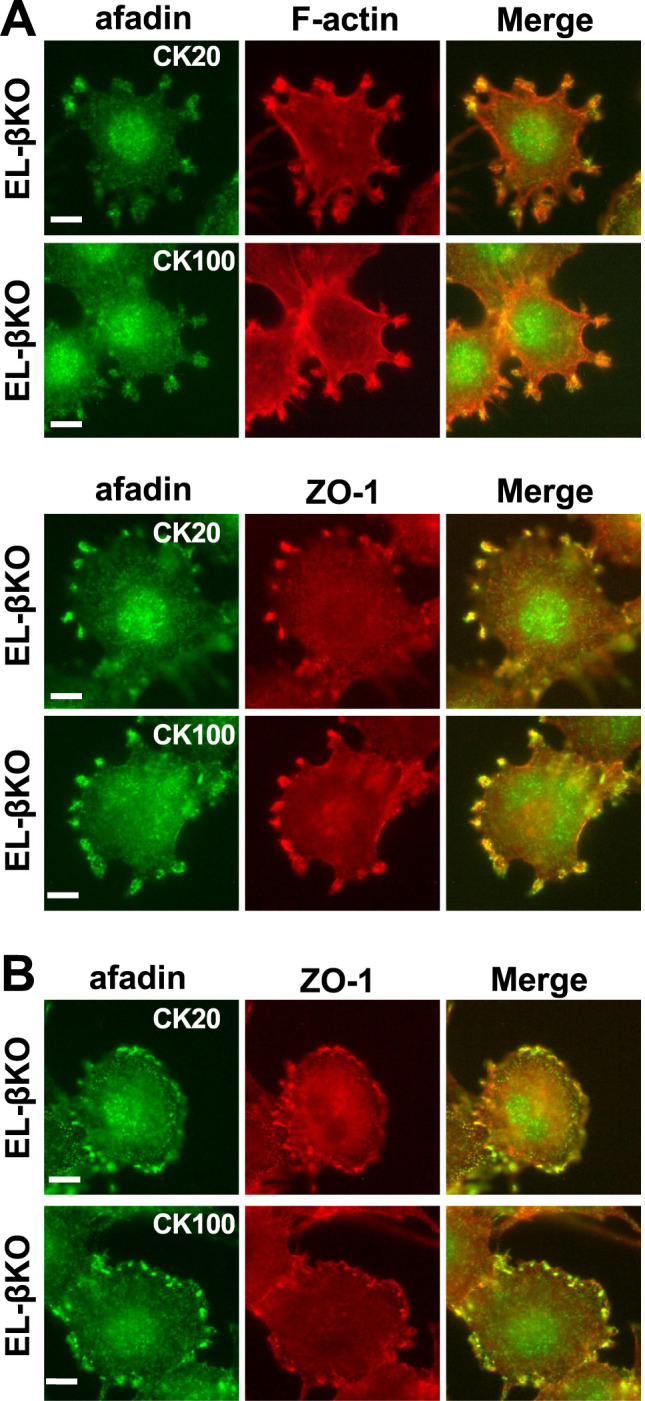
Localization of afadin, ZO-1, and actin filaments in EL-βKO cells treated with CK666 Immunostaining of EL-βKO cells treated with 20 μM or 100 μM CK666 (CK20 and CK100, respectively) for 30 minutes. (A) Cells exhibiting lamellipodia-like structures. Upper panels: green, afadin; red, actin filaments (F-actin). Lower panels: green, afadin; red, ZO-1. (B) Cells lacking lamellipodia-like structures. Green, afadin; red, ZO-1. Afadin and ZO-1 were localized at the cell periphery.

Since two actin polymerization inhibitors, CD and LB, severely affected the localization of afadin, ZO-1, and actin filaments, we next examined the effect of an Arp2/3 complex inhibitor, CK666, on their localization (Fig. 5). When EL-βKO cells were treated with 20 μM or 100 μM of CK666, actin filament aggregates were not prominent (Supplementary Fig. 2D and E). However, we did observe various alterations in lamellipodia-like structures and in the localization of afadin and ZO-1. In some cases, lamellipodia-like structures resembled those in untreated cells, and afadin and ZO-1 localized at these structures (Fig. 5A). In other cases, the lamellipodia-like structures disappeared, and localization of afadin and ZO-1 was observed at the cell periphery (Fig. 5B). Unexpectedly, different doses of CK666 did not result in distinct localization changes of afadin and ZO-1, unlike those observed after CD or LB treatment. Instead, the localization patterns of these molecules appear to be influenced stochastically or through unidentified mechanisms. These findings suggest that although the lamellipodia-like structures are not typical lamellipodia, they are influenced by CK666 treatment. Moreover, the localization of afadin and ZO-1 is affected in a heterogenous manner.

### The localization of afadin and ZO-1 in αD and BPD cells

**Fig. 6:**
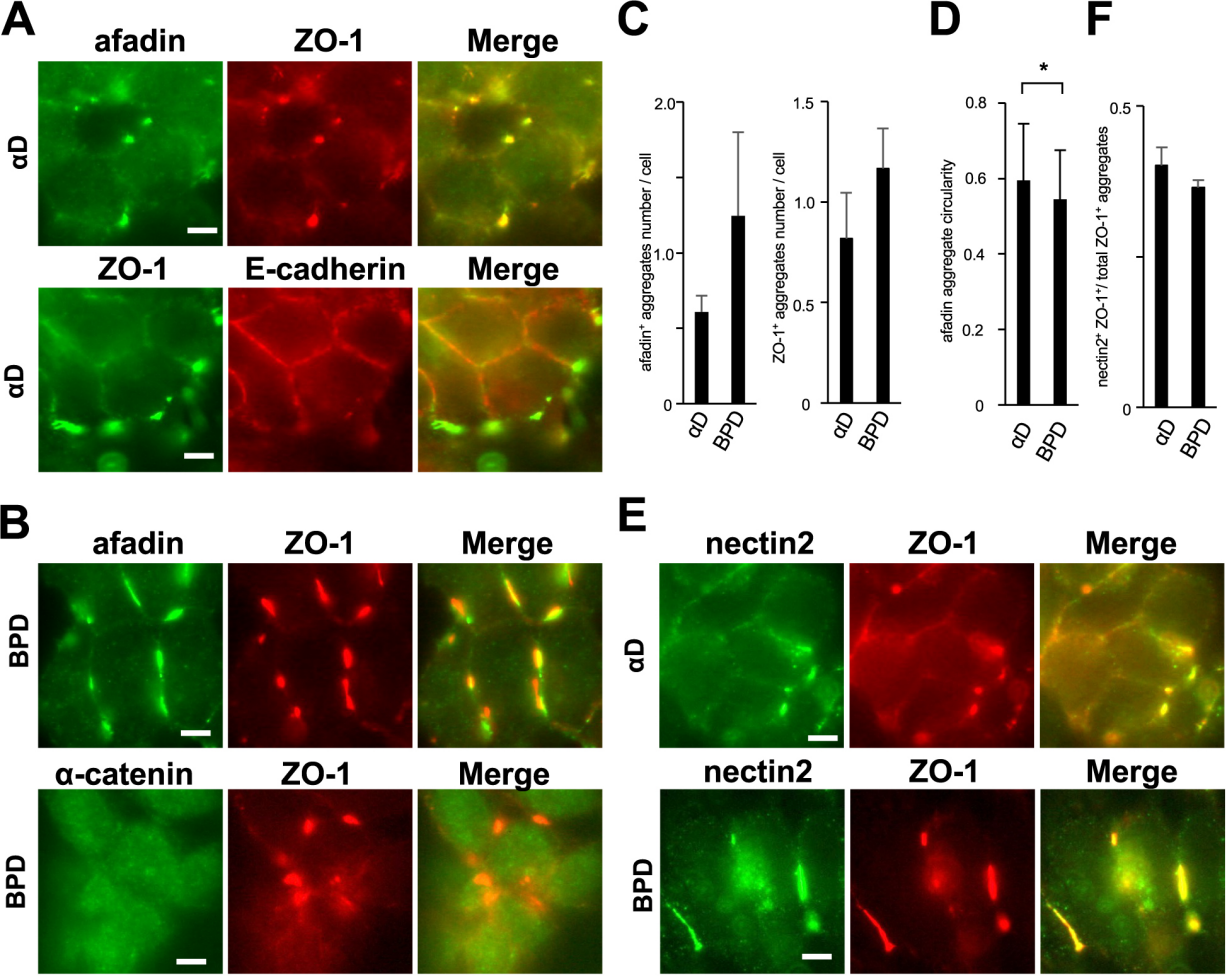
Localization of afadin and ZO-1 in αD and BPD cells (A) Immunostaining of αD cells. Upper panels: green, afadin; red, ZO-1. Lower panels: green, ZO-1; red, E-cadherin. Afadin and ZO-1 formed aggregates at the cell-cell contact sites, whereas E-cadherin was continuously localized. Scale bar = 5 μm. (B) Immunostaining of BPD cells. Upper panels: green, afadin; red, ZO-1. Lower panels: green, α-catenin; red, ZO-1. Scale bar = 5 μm. (C) Quantification of the number of afadin-positive (left) and ZO-1-positive (right) aggregates per cells. Error bars represent SD. (D) Quantification of the Crofton circularity of individual afadin aggregates. Circularity values were calculated on a per-particle basis using the Crofton method. The mean circularity was significantly lower in BPD cells (*p<0.05, Mann-Whitney U test). Error bars represent SD. (E) Immunostaining for nectin-2 (green) and ZO-1 (red). Scale bar = 5 μm. (F) Proportion of nectin-2/ZO-1 double-positive aggregates among total ZO-1-positive aggregates. Error bars represent SD.

To clarify the role of CCC components in the localization of afadin and ZO-1 in F9-derived cells, we examined their distribution in αD cells and BPD cells. In αD cells, afadin and ZO-1 formed aggregates at cell-cell contact sites without accompanying actin filament condensation (Fig. 6A and E). In contrast, junctional E-cadherin-β-catenin complex exhibited a linear localization at cell-cell contact sites (Fig. 6A). Although ZO-1 was not excluded from these linear cell-cell contact regions, it did not show clear condensation (Fig. 6A). Next, using BPD cells, we investigated the role of β-catenin in afadin/ZO-1 aggregate formation and whether endogenous free α-catenin colocalizes with these aggregates. Even in BPD cells, afadin/ZO-1 aggregates were observed (Fig. 6B and E). Within these aggregates, endogenous α-catenin signals were barely detectable (Fig. 6B). Unexpectedly, in BPD cells, afadin/ZO-1 aggregates were observed more frequently than in αD cells. Moreover, large, continuous linear aggregates were also observed. (Fig. 6A–E). Thus, loss of alpha-catenin and beta-catenin had different effects in F9-derived cells, whereas such differences were not observed in EL-derived cells. It has been reported that nectin-2, a direct binding partner of afadin, is highly expressed in F9 cells but not in EL cells. Therefore, we examined the localization of nectin-2 in αD and BPD cells. As expected, nectin-2 colocalized with afadin/ZO-1 aggregates in both cell lines (Fig. 6E and F). Moreover, in αD cells, weak signals of nectin-2 were also detected at cell-cell contact sites (Fig. 6E). These results indicate that afadin/ZO-1 aggregates form in αD and BPD cells and that endogenous α-catenin does not colocalize with these aggregates. Our findings further suggest that nectin-2, which is expressed in F9 cells, plays a role in afadin/ZO-1 aggregate formation, which does not occur in EL-derived cell. Additionally, our data suggest that the endogenous junctional E-cadherin-β-catenin complex influences both the figure of afadin/ZO-1 aggregates and the localization of nectin-2.

### The roles of afadin and ZO-1 in afadin/ZO-1 aggregate formation in F9-derived cells

**Fig. 7:**
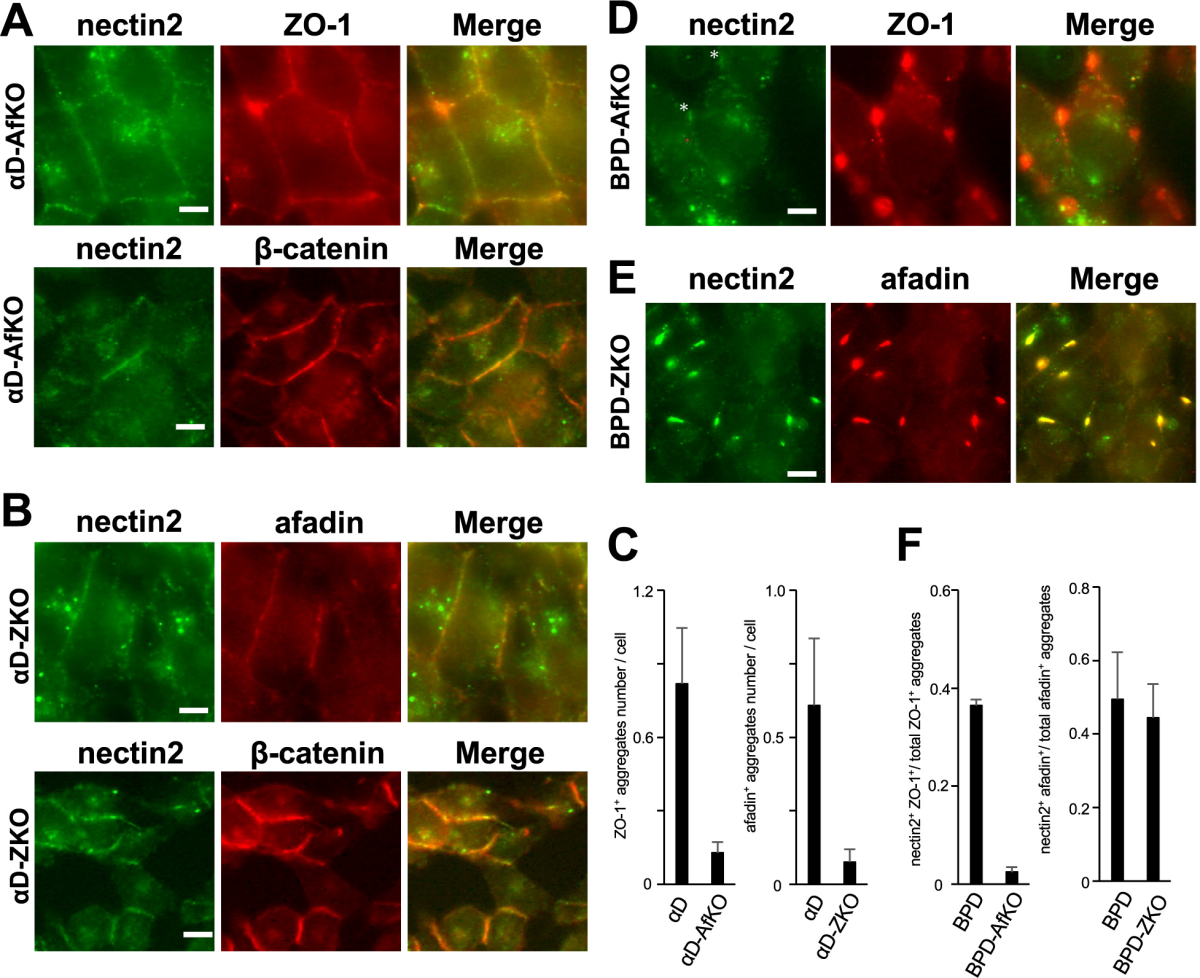
Localization of afadin, ZO-1, and nectin-2 in afadin-deficient or ZO-1-deficient αD and BPD cells (A) Immunostaining of αD-AfKO cells. Upper panels: green, nectin-2; red, ZO-1. Lower panels: green, nectin-2; red, β-catenin. Scale bar = 5 μm. (B) Immunostaining of αD-ZKO cells. Upper panels: green, nectin-2; red, afadin. Lower panels: green, nectin-2; red, β-catenin. Scale bar = 5 μm. (C) Quantification of the number of ZO-1-positive (left) and afadin-positive (right) aggregates per cell in αD, αD-AfKO, αD-ZKO cells. Error bars represent SD. (D, E) Immunostaining of BPD-AfKO cells (D) and BPD-ZKO cells (E). Green, nectin-2; red, ZO-1 and afadin, respectively. Scale bar = 5 μm. Weak nectin-2 signals in D (asterisks) were observed due to cross-reactivity of the anti-rat secondary antibody with the mouse anti-ZO-1 antibody. Since these signals were too weak to be recognized as positive (Supplementary Fig. 4), the double staining data in Fig. 6E, 7A, and 7D are therefore considered valid. (F) Proportion of nectin-2/ZO-1 double-positive aggregates among total ZO-1-positive aggregates (left) and nectin-2/afadin double-positive aggregates among total afadin-positive aggregates (right). Error bars represent SD.

Then, we investigated whether afadin or ZO-1 is required for the formation of afadin/ZO-1 aggregate in F9-derived cells (Fig. 7). In αD-AfKO and αD-ZKO cells, ZO-1 and afadin aggregates were barely detectable, respectively (Fig. 7A–C). Instead, in these cells, ZO-1 and afadin were localized at cell-cell contact sites, where not only junctional E-cadherin-β-catenin complex but also nectin-2 were present (Fig. 7A and B). In contrast, in both BPD-AfKO and BPD-ZKO cells, ZO-1 aggregates and afadin aggregates were frequently observed, respectively (Fig. 7D and E, and Supplementary Fig. 3). In these cells, nectin-2 colocalized with afadin aggregates but not with ZO-1 aggregates (Fig. 7D, E and F). Since our anti-rat second antibody for the nectin-2 weakly cross-reacts with mouse anti-ZO-1 antibody, we consider the weak nectin-2 signals at ZO-1 aggregates as negative in nectin-2 and ZO-1 double staining samples (Fig. 7D). These results indicate that ZO-1 and afadin are required for the formation of afadin or ZO-1 aggregates in αD-derived cells, but not in BPD-derived cells. Furthermore, our findings suggest that in BPD-derived cells, nectin-2 is involved in the formation of afadin aggregates but not of ZO-1 aggregates.

### The roles of nectin-2 in afadin/ZO-1 aggregate formation in F9-derived cells

**Fig. 8:**
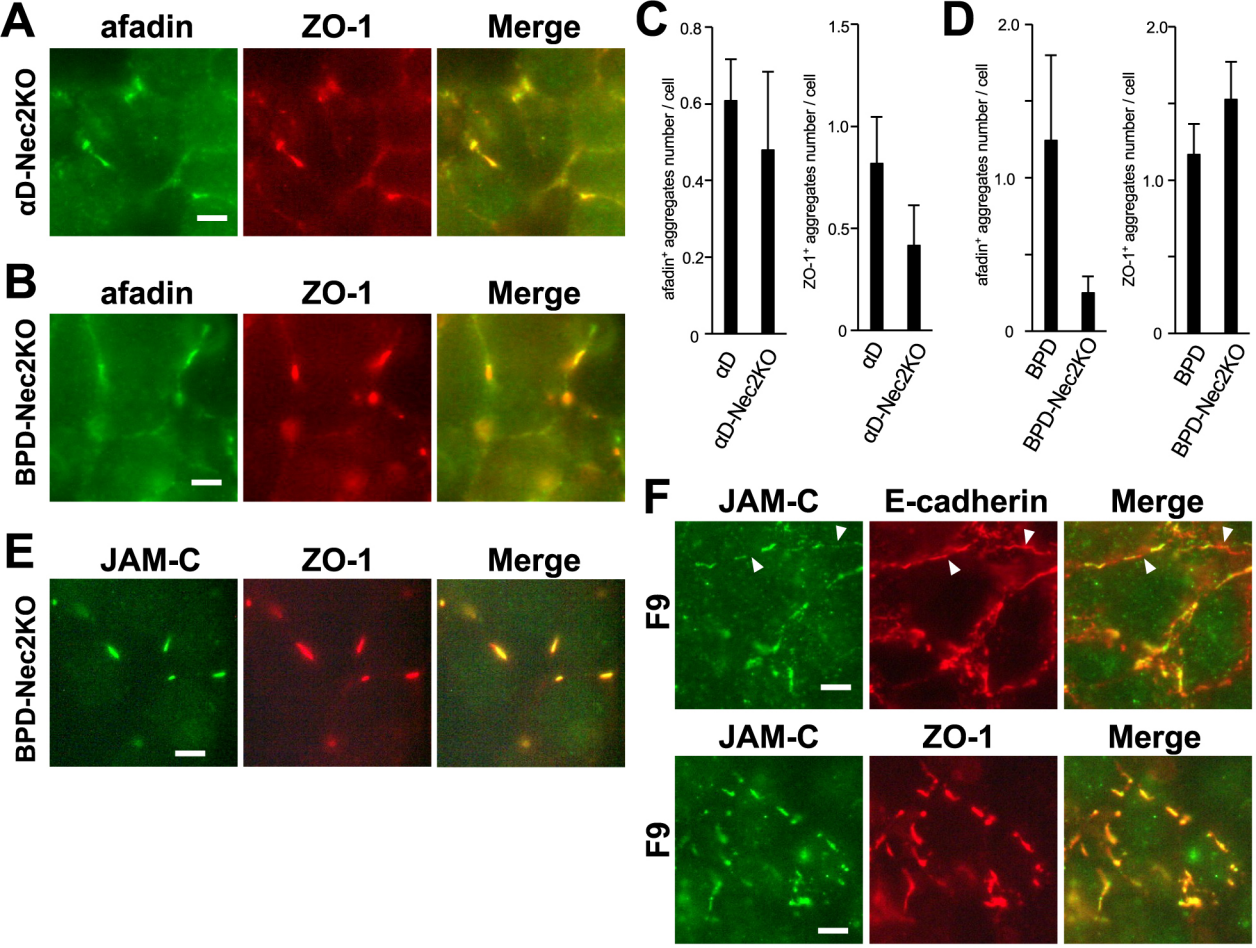
Localization of afadin, ZO-1, E-cadherin, and JAM-C in nectin-2-deficient αD and BPD cells, and F9 cells (A) Immunostaining of αD-Nec2KO cells. Green, afadin; red, ZO-1. Scale bar = 5 μm. (B) Immunostaining of BPD-Nec2KO cells. Green, afadin; red, ZO-1. Scale bar = 5 μm. (C, D) Quantification of the number of afadin-positive aggregates (left) and ZO-1-positive aggregates per cell in αD-Nec2KO cells (C) and BPD-Nec2KO cells (D). Data for αD and BPD are the same as in Fig. 6. Error bars represent SD. (E) Immunostaining of BPD-Nec2KO cells. Green, JAM-C; red, ZO-1. Scale bar = 5 μm. (F) Immunostaining of F9 cells. Upper panels: green, JAM-C; red, E-cadherin. Lower panels; green, JAM-C; red, ZO-1. Scale bar = 5 μm. In F9 cells, JAM-C was not necessarily colocalized with E-cadherin (arrowheads).

To investigate the role of nectin-2 in the localization of afadin and ZO-1 in F9-derived cells, we examined their distribution in two nectin-2-deficient cell lines, αD-Nec2KO and BPD-Nec2KO. Unexpectedly, even in the absence of nectin-2, aggregates of afadin and ZO-1 were observed in both αD-Nec2KO and BPD-Nec2KO cells, although quantitative analysis revealed a significant decrease in the number of afadin-positive aggregates in BPD-Nec2KO cells compared with BPD cells (Fig. 8A–D). In BPD-Nec2KO, afadin signals were frequently detected within ZO-1 aggregates. However, they were weak, and afadin was also observed at cell-cell contact sites surrounding the ZO-1 aggregates. As a result, the afadin signals could not be classified as “aggregates” according to the parameters used in this study. Notably, ZO-1 aggregates were frequently formed even in the absence of nectin-2. Moreover, as shown above, nectin-2 did not colocalize with ZO-1 aggregates in the absence of afadin. Taken together, these findings suggest that a ZO-1-binding membrane protein may contribute to the formation of afadin/ZO-1 aggregates. JAMs, which are ZO-1-binding membrane proteins, are strong candidates. Among them, JAM-C has been reported to be expressed in F9 cells and to colocalize with E-cadherin (Satohisa *et al.*, 2005). Because the previously reported anti-JAM-C antibody was not available to us, we generated our own anti-JAM-C antibody and examined JAM-C localization (Supplementary Fig. 4). As expected, JAM-C colocalized with afadin/ZO-1 aggregates in BPD-Nec2KO cells (Fig. 8E). In parental F9 cells, JAM-C was partially, but not completely, colocalized with E-cadherin and showed strong colocalization with ZO-1 at cell-cell contact sites (Fig. 8F). These results indicate that JAM-C is a component of the cadherin-based cell-cell contact sites in non-epithelial F9 cells. Our findings also suggest that nectin-2 and JAM-C redundantly support afadin/ZO-1 aggregate formation in the absence of α- and β-catenin in F9 cells.

### Afadin/ZO-1 aggregate formation in the presence of 1,6-HD

Recently, it has been reported that phase separation is involved in the aggregate formation of afadin and ZO-1. To determine whether this also applies to BPD and BPD-derived cells, we examined the localization of afadin and ZO-1 in these cells treated with 1,6-HD. Following treatment, in all cells including BPD, BPD-ZKO, BPD-AfKO and BPD-Nec2KO, afadin and/or ZO-1 aggregates remained visible (Supplementary Fig. 5). These findings suggest that phase separation may not be essential for the formation of afadin and/or ZO-1 aggregates.

## Discussion

In this study, we investigated the localization of afadin, ZO-1 and actin filaments using various junction-deficient non-epithelial cells. As a result, we identified previously unrecognized and cryptic aspects of their interactions, which are normally masked by the presence of their major binding partners in junctions, such as α-catenin and claudin. Notably, the formation of afadin/ZO-1 aggregates in F9-derived cells suggests complex and cryptic interplay between afadin, ZO-1, and their respective membrane-associated partners. Here, we discuss the roles of actin filament organization in the localization of afadin and ZO-1. We also discuss the complex interactions of afadin and ZO-1 with their respective membrane partners and with the junctional E-cadherin-β-catenin complex.

### Effect of CD, LB, and CK666 treatments on the localization of afadin, ZO-1, and actin filaments

To date, the effects of CD and LB on the localization of afadin and/or ZO-1 have been reported in epithelial cells expressing CCC (Stevenson and Begg, 1994; Yamada *et al.*, 2004). In those systems, it is difficult to distinguish whether the observed localization changes are due to alterations in actin polymerization or to changes in TJs and AJs. The treatment of EL-αKO or EL-βKO cells with CD, LB, and CK666 allows us to reconsider the roles of actin-based cytoskeleton in cell-cell junction formation (Table 2). Although both afadin and ZO-1 are actin-binding proteins, they primarily colocalize with actin filaments at cell-cell contact sites but not with other structures of actin filament. This suggests that the interaction of afadin and ZO-1 with actin filaments is regulated by unknown mechanisms. We observed that, in EL-αKO and EL-βKO cells, afadin and ZO-1 generally do not colocalize with actin filaments except for the co-localization of afadin and ZO-1 with actin filaments at lamellipodia-like structures. Because this localization pattern of ZO-1 was also observed in parental EL cells (Fig. 2C), and ZO-1 localization at the lamellipodia of wound edges in fibroblasts has been reported previously (Taliana *et al.*, 2005), such localization appears to be a common feature of ZO-1. When these cells were treated with relatively high concentrations of LB or CD, different types of actin filament structures, referred to as “actin filament aggregates”, emerged. ZO-1 aggregates colocalized with these actin filament aggregates, and afadin aggregates were often found adjacent to the ZO-1/actin aggregates. Such adjacent localization of afadin and ZO-1 aggregates was also recently reported in epithelial MDCK cells treated with LB (Kuno *et al.*, 2025). Additionally, when cells were treated with LB at a very low concentration or CK666, actin filament aggregates were not prominent. In these cell, ZO-1 preferentially colocalized with actin fibers in lamellipodia-like structures. Moreover, following CK666 treatment, small aggregates of afadin and ZO-1 were frequently detected at the cell periphery, where lamellipodia-like structures were not observed. These localization changes in afadin and ZO-1 may seem to be merely artifact. However, we propose that these findings suggest the possibility that the status of actin polymerization influences junction formation by regulating afadin and ZO-1 localization. In addition to the outside-in signals from junctional complex formation that regulate fasciculation of actin filaments, we should also consider the inside-out signals from status of actin filaments that regulate junctional complex formation.

### Aggregate formation of afadin and ZO-1

Aggregates of ZO-1 observed in BPD-Nec2KO cells highlighted the involvement of JAM-C, a ZO-1-binding membrane proteins previously unrecognized in non-epithelial junction. In F9 cells, however, JAM-C has been reported to be expressed and to colocalize with E-cadherin (Satohisa *et al.*, 2005). In our observations, JAM-C partially colocalizes with E-cadherin and precisely colocalizes with ZO-1. Previously, we reported the presence of two types of E-cadherin-based cell contact sites, those with and without ZO-1, in F9 cells. Further investigation is needed to elucidate the molecular architecture and functional significance of these two distinct cadherin-based junctions in non-epithelial F9 cells. In addition, it should be noted that afadin was localized not only at ZO-1 aggregates but also at the surrounding cell-cell contact sites in BPD-Nec2KO cells. This finding suggests the presence of a nectin-2-independent membrane-tethering mechanism for afadin in F9-derived cells, which has not been reported so far.

**Fig. 9:**
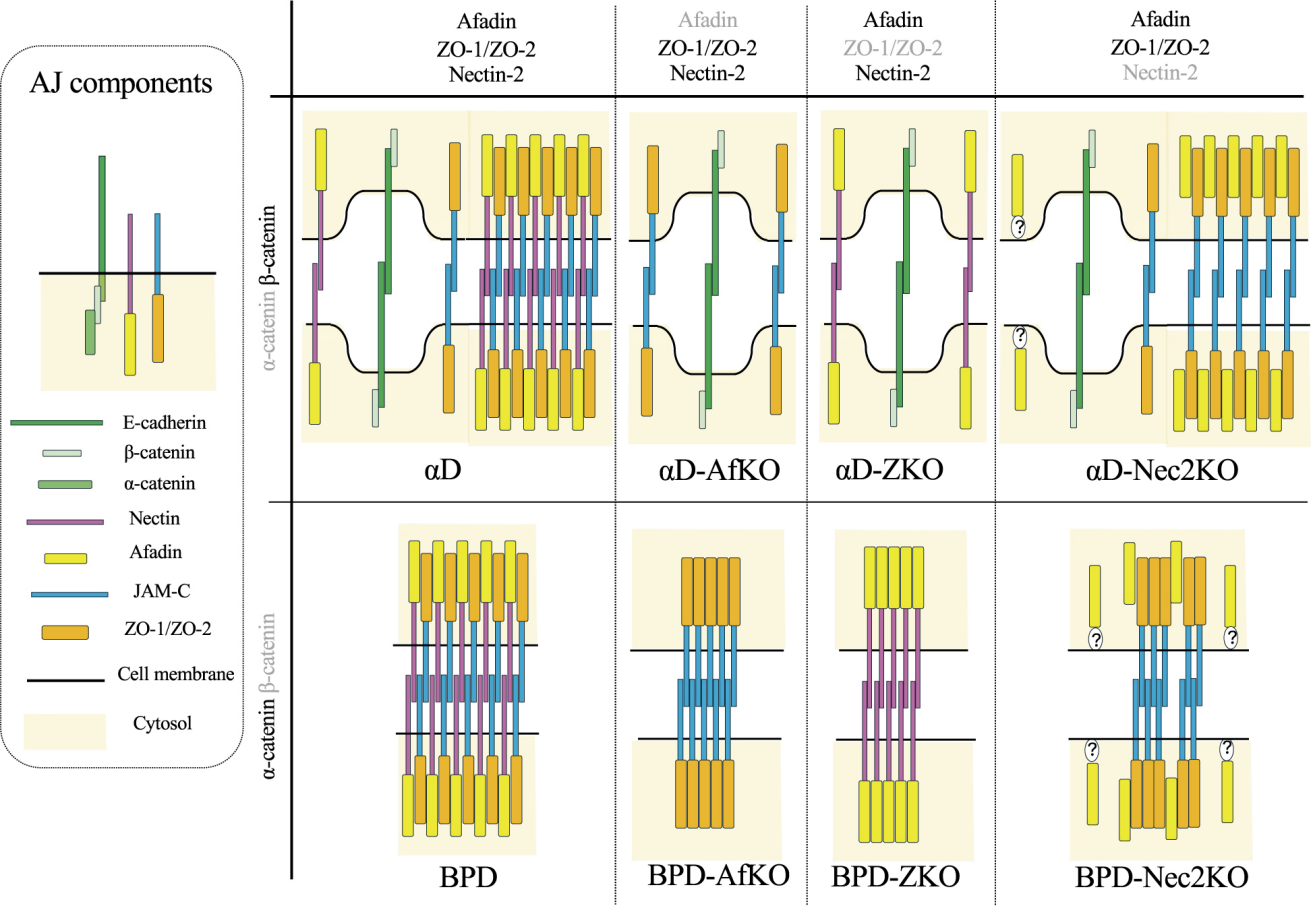
Schematic model of afadin/ZO-1 aggregates in the absence of CCC components The diagrams illustrate the molecular architectures of afadin/ZO-1 aggregates in αD, BPD, and their derivative cell lines. The left panel shows the color-coded components of AJs. For simplicity, ZO-2 is not shown. The nectin-2-afadin complex and the JAM-C-ZO-1 complex form stable aggregates even in the presence of the E-cadherin-β-catenin complex (αD and BPD). Each complex independently forms aggregates in the absence of the E-cadherin-β-catenin complex (BPD-AfKO and BPD-ZKO), but not in its presence (αD -AfKO and αD -ZKO). In the absence of nectin-2, the JAM-C-ZO-1 complex forms stable aggregates even when the E-cadherin-β-catenin complex is present (αD -Nec2KO). When both nectin-2 and the E-cadherin-β-catenin complex are absent, the JAM-C-ZO-1 complex still forms aggregates with afadin, although the amount of afadin is lower than that in the presence of nectin-2. In this case, afadin is tethered to the plasma membrane through an unidentified mechanism.

Aggregates of afadin and/or ZO-1 observed in BPD, BPD-AfKO, BPD-ZKO, and BPD-Nec2KO suggest a complex and cryptic interplay among these proteins (Fig. 9). Although afadin and ZO-1 were also colocalized at cell-cell contact sites in parental F9 cells, the presence of α-catenin, which binds to both afadin and ZO-1, may have masked their direct interaction. Recently it has been reported that phase separation is involved in afadin and ZO-1 aggregate formation. However, it seems not to be mainly involved in the aggregate formation in BPD-derived cells, since these aggregates formed even in cells treated with 1,6-HD. Because the commonly used functional concentration of 1,6-HD (5%) was toxic to our cells, further investigation will be required to draw a definitive conclusion on this point. The presence of ZO-1 and afadin aggregates in BPD-AfKO, BPD-ZKO cells, respectively, indicates that nectin-2-afadin and JAM-C-ZO-1 complexes can independently form aggregates, likely through trans-interactions of membrane proteins and the self-association of afadin or ZO proteins. In BPD cells, afadin and ZO-1 largely colocalized, and distinct afadin or ZO-1 aggregates were rarely observed. These findings suggest that nectin-2-afadin and JAM-C-ZO-1 complexes preferentially interact with each other rather than undergoing self-interaction. One possibility is that afadin, bound to nectin-2, and ZO-1, bound to JAM-C, preferentially interact with each other. Another possibility is that trans-interactions of nectin-2 promote those of JAM-C, and vice versa, despite the difference in the number of Ig folds, three in nectin-2 and two in JAM-C. In BPD-Nec2KO cell, although intensity of afadin signals was decreased, afadin still colocalized with ZO-1 aggregates. This finding suggests that, even in the absence of nectin-2, afadin interact with JAM-C-ZO-1 complexes, overcoming their self-association. These regulatory dynamics, which extend beyond simple protein-protein interactions, may represent a cryptic mechanism underlying the structural organization of cell-cell junctions.

It is interesting that the number and figure of aggregates are different in αD cells and in BPD cells. Moreover, these aggregates disappear in αD cells but persist in BPD cells in the absence of afadin or ZO-1 expression. The main difference between αD and BPD cells is the expression of E-cadherin, which is complexed with β-catenin in αD cells but not in BPD cells (Fukunaga *et al.*, 2005; Maeno *et al.*, 1999). In αD cells, a portion of nectin-2 colocalizes with E-cadherin at cell-cell contact sites. In αD cells lacking afadin or ZO-1 expression, most of nectin-2 colocalizes with E-cadherin at these sites. Although the junctional E-cadherin-β-catenin complex lacks cytoplasmic binding partners in the absence of α-catenin, its extracellular domain could be involved in trans-interaction(Harrison *et al.*, 2011). Extracellular domain of E-cadherin contains five cadherin repeats which form relatively rigid structure in the presence of calcium ions. Trans-interaction of E-cadherin extracellular domain could alter the physical state of membrane contact sites, thereby hindering nectin-2 and JAM-C from participating in the formation of afadin and ZO-1 aggregates. In the presence of junctional E-cadherin-β-catenin complex, the co-expression of both afadin and ZO-1 is required for synergistic aggregate formation with nectin-2 and JAM-C. This is consistent with afadin/ZO-1 aggregate formation mechanisms discussed above. This also could explain the reduced number of afadin/ZO-1 aggregates in αD cells and the disappearance of aggregate in αD cells lacking afadin or ZO-1 (Fig. 9). It is notable that the number of afadin/ZO-1 aggregates was not reduced in αD-Nec2KO cells. In this case, the junctional E-cadherin-β-catenin complex may support afadin/ZO-1 aggregate formation even in the absence of nectin-2. A previous study in *C. elegans* reported that cadherin expression but not that of α-catenin is genetically linked to an afadin-dependent phenotype (Lockwood *et al.*, 2008). This finding could also be interpreted as a consequence of trans-interaction involving the cadherin-β-catenin complex. These possibilities should be elucidated.

In this study, we carefully examined the localization of afadin, ZO-1 and actin filaments in the absence of AJs and TJs and identified three novel aspects of their interactions. First, we found that changes in the polymerization state of actin filaments affects the localization of afadin and ZO-1. This suggests the presence of an inside-out signal mechanism, where junctions involving afadin and ZO-1 are influenced by the regulation of actin polymerization. Second, nectin-2 and JAM-C independently and cooperatively regulate the localization of afadin and ZO-1, respectively. Third, the junctional E-cadherin-β-catenin complex, but not α-catenin, affects the localization of afadin and ZO-1. These findings provide important insights into the complex regulatory mechanisms underlying junction formation.

## Data Availability

All data in this study are included in the main article or in the supplementary materials.

## Author Contributions

YN, SU, MK, and AN established the cell lines. YN, MK, and AN constructed the pCAS9-PAC plasmid. YN, SU, MK, HN, TI, KH, HM, CK, and AN constructed the vectors. YK and MF produced and characterized anti-JAM-C pAbs. SU and AN performed the image analysis. MK and AN conducted the Western blot analysis. SU performed the statistical analysis. AN conceived and designed the experiments and wrote the manuscript.

## Conflict of Interest

The authors declare no competing interests.

## Figures and Tables

**Table 1:** List of cell lines used in this study

Cell Name	Description
EL cells	E-cadherin-expressing L cells
EL-αKO cells	α-catenin-deficient EL cells
EL-βKO cells	β-catenin-deficient EL cells
F9 Cells	Mouse teratocarcinoma cells
αD cells	α-catenin-deficient F9 cells
αD-AfKO cells	Afadin-deficient αD cells
αD-ZKO cells	ZO-1/ZO-2-deficient αD cells
αD-Nec2KO cells	Nectin-2-deficient αD cells
BPD cells	β-catenin/plakoglobin-deficient F9 cells
BPD-AfKO cells	Afadin-deficient BPD cells
BPD-ZKO cells	ZO-1/ZO-2-deficient BPD cells
BPD-Nec2KO cells	Nectin-2-deficient BPD cells

**Table 2:** Summary of the localization of afadin, ZO-1, and actin filaments in EL-βKO cells treated with CD, LB, and CK666

Inhibitor	Concentration	Afadin	ZO-1	F-actin
—		At lamellipodia-like structures	At lamellipodia-like structures and in the cytoplasm	
CD	1 μM	In the cytoplasm, adjacent to ZO-1/Actin aggregates	In the cytoplasm, high-level co-localization with actin aggregates	In the cytoplasm (Large aggregates)
	0.25 μM			In the cytoplasm (Small aggregates)
LB	0.1 μM			
	0.025 μM	At lamellipodia-like structures		
CK666	100 μM	At lamellipodia-like structures and at the cell periphery.		
	20 μM			

## Abbreviations

TJ: tight junction
AJ: adherens junction
CCC: cadherin-catenin complex
CD: cytochalasin D
LB: latrunculin B
mAb: monoclonal antibody
pAb: polyclonal antibody
1,6-HD: 1,6-Hexanediol (Hexamethylene Glycol)
