# Supplementary material for: Context-dependent interactions among afadin, ZO-1, and actin filaments
Source: Cell Struct Funct. 2025 Nov 18;50(2):223–38. doi: 10.1247/csf.25019 (PMC12967520; doi:10.1247/csf.25019)
Supplement: Supplementary file 1 — Supplementary Materials [file csf_50_25019_1.pdf]

Supplementary Materials and Method

Validation of JAM-C antibody specificity

A mammalian expression vector of mouse JAM-C was constructed as follows. Total RNA was extracted from the mouse brain, and cDNA was synthesized using Superscript III Reverse Transcriptase (Thermo Fisher Scientific). The full-length mouse JAM-C cDNA was amplified by PCR using the synthesized cDNA as a template, with the following primer pair: forward 5'-cggctcgagATGGCGCTGAGCCGGCGGCTGCGA-3' and reverse 5'-cgcagatctTCAGATAACAAAGGACGATTTGTG-3'. The resulting PCR products was digested with Xho I and Bgl II, and subcloned into the pCAGGS mammalian expression vector(Niwa *et al.*, 1991). Correct PCR amplification of JAM-C cDNA was verified by DNA sequencing.

To obtain fibroblasts expressing mouse JAM-C, mouse L cells were transfected with the mouse JAM-C-expression vector. Stable transfectant clones were selected in medium containing 500 μg/ml of G418 (Nacalai Tesque, Japan).

1,6-HD treatment

For 1,6-Hexanediol (1,6-HD; also known as Hexamethylene Glycol), cells were incubated 2% 1,6-HD for 5 minutes. When the concentration exceeded 2.5%, cells on the coverslip completely disappeared.

Binarization of images

In EL-derived cells, RGB images were converted to grayscale by channel averaging, and background signals were estimated and subtracted using a Gaussian filter (σ = 10), followed by normalization to a 0–1 range by dividing by the maximum value. Depending on the background characteristics of each antibody, the thresholding method was selected: Afadin and α-catenin were binarized using the Yen method (threshold_yen in scikit-image), and ZO-1 and Actin were binarized using the Otsu method (threshold_otsu). Watershed segmentation was applied when necessary. Afadin, α-catenin, and ZO-1 were defined as particles if they contained at least one pixel, whereas actin was defined as particles if the area was ≥10 pixels with a Crofton circularity ≥0.7.

In F9-derived cells, individual fluorescence channels were extracted using FIJI (Split Channels). Background subtraction was performed with a rolling ball radius of 50 pixels (RB50). Exposure time differences were corrected by calculating a correction factor based on the Mean Gray Value (MGV). Binarization was performed using the MaxEntropy method, in which a representative image was selected for each experiment to determine the threshold,

and the same threshold was uniformly applied to all images within that experiment. Watershed segmentation was then applied to separate adjacent regions. For all antibodies, aggregates were defined as connected regions of ≥1 pixel, without applying circularity constraints.

Colocalization analysis

Binarized images from different channels were compared on a pixel-by-pixel basis, and colocalization was defined when signal regions overlapped. For each ROI, the number of overlapping pixels was quantified, and the presence and proportion of colocalization were calculated. Colocalization values were obtained on a per-image basis, and these image-level values were used for statistical comparison across groups. Results were saved as CSV files, and labeling images were generated for visual verification. Statistical significance was evaluated using the Mann–Whitney U test. Data are presented as mean ± SD, and p-values < 0.05 were considered statistically significant.

Actin aggregate density analysis

The number of Actin particles was calculated according to the criteria described above. To correct for variations in cell density across images, the Actin channel was preprocessed with CLAHE (clip limit = 2.0, tile grid = 8×8) and a Gaussian filter ($\sigma = 2.0$), followed by Otsu thresholding to generate cell area masks. Morphological closing (disk radius = 7 pixels), removal of small objects (<500 pixels), and hole filling (<20,000 pixels^2) were applied to refine the masks. The cell-covered area was converted to μm^2 using the pixel size, and Actin aggregate density was expressed as the number of particles per μm^2. Aggregate density values were obtained on a per-image basis, and these image-level values were used for statistical comparison across groups.

Distance analysis

For geometric analysis, Actin particles were set as the reference, and Afadin–Actin pairs or ZO-1–Actin pairs were evaluated as the target combinations. Distance was defined as the minimum distance between the boundary pixels of reference and target particles and converted to μm using a pixel size of 0.11233 μm/px. Distance analyses were performed on a per-particle basis, and statistical significance was evaluated using the Mann–Whitney U test. Results are presented as distribution plots (distance).

Line scan analysis

Line scan analysis was performed using Fiji. A straight line was drawn perpendicular to the cell-cell contact sites, and fluorescence intensity profiles were obtained with the "Plot Profile"

function.

Supplementary Figure 1

Localization of α-catenin and β-catenin in EL-αKO and EL-βKO cells

Immunostaining of co-cultured EL-αKO and EL-βKO cells; green: α-catenin, red: β-catenin. Scale bar = 20 μm.

Supplementary Figure 2

Effects of CD and LB on the expression of afadin and ZO-1 and the localization of actin filaments

(A) Western blot analysis of afadin and ZO-1 in untreated EL-βKO cells (Control), EL-βKO cells treated with 1 μM CD for 15 minutes (CD1), and EL-βKO cells treated with 2 μM LB (LB2) for 1 hour. (B) Immunostaining of EL-βKO cells treated with 1 μM or 0.25 μM CD for 15 minutes; red: F-actin. Scale bar = 10 μm. (C) Immunostaining of EL-βKO cells treated with 2 μM, 0.1 μM, or 0.025 μM LB for 1 hour; red: F-actin. Scale bar = 10 μm. (D) Western blot analysis of afadin and ZO-1 in untreated EL-βKO cells (Control), EL-βKO cells treated with 100 μM CK666 for 30 minutes (CK100). (E) Immunostaining of EL-βKO cells treated with 20μM or 100 μM CK666 (CK 100 μM and CK 20μM, respectively) for 30 minutes; red: F-actin. Scale bar = 10 μm.

Supplementary Figure 3

The number of aggregates in αD-derived cells

Quantification of the number of ZO-1-positive (left) and afadin-positive (right) aggregates per cell in BPD, BPD-AfKO, and BPD-ZKO cells. Error bars represent SD.

Supplementary Figure 4

Validation of anti-rat Ig secondary antibody

(A) Immunostaining of BPD-Nec2KO cells. Cells were stained either without the anti-nectin-2 antibody (upper panels) or without the anti-ZO-1 antibody (lower panels). Green, anti-rat Ig (upper panel) and nectin-2 (lower panel); red, ZO-1. Even in the absence of the anti-nectin-2 antibody and cell surface nectin-2, anti-rat secondary antibody showed weak signals when the anti-ZO-1 antibody was present. In the absence of the anti-ZO-1 antibody, the anti-rat secondary antibody combined with the anti-nectin-2 antibody did not produce detectable signals. (B) Immunostaining of co-cultured BPD-AfKO and BPD-ZKO cells. Green, nectin-2; red, ZO-1. White and magenta asterisks indicate BPD-ZKO and BPD-AfKO cells, respectively. Allows indicate the regions used for line scan analysis. Scale bar = 10 mm. (C) Line scan profiles showing the distribution of nectin-2 (or anti-rat secondary antibody) signal intensity in the regions indicated in (B). Left, BPD-ZKO; right, BPD-AfKO.

Supplementary Figure 5

Reactivity of the anti-JAM-C antibody

Immunostaining of L cells and L cells transfected with the JAM-C expression vector (JAM-C-L) using the anti-JAM-C antibody.

Supplementary Figure 6

Localization of afadin and ZO-1 in cells treated with 1,6 HD.

Immunostaining of BPD cells (A), BPD-ZKO cells (B), BPD-AfKO cells (C), and BPD-Nec2KO cells (D) treated with 2% 1,6-HD for 5 minutes. Upper panels: untreated control cells (Control); lower panels: 1,6-HD-treated cells. (A and D) Green, afadin; red, ZO-1. (B and C) Green, afadin and ZO-1, respectively.

Supplementary Table 1

Guide sequences and PCR primers used for CRISPR/Cas9-mediated gene disruption and its validation.

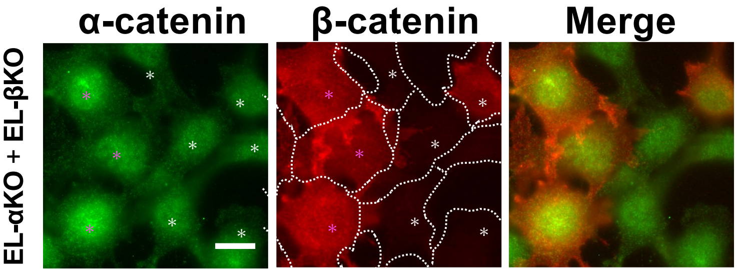

Supplementary Figure 1

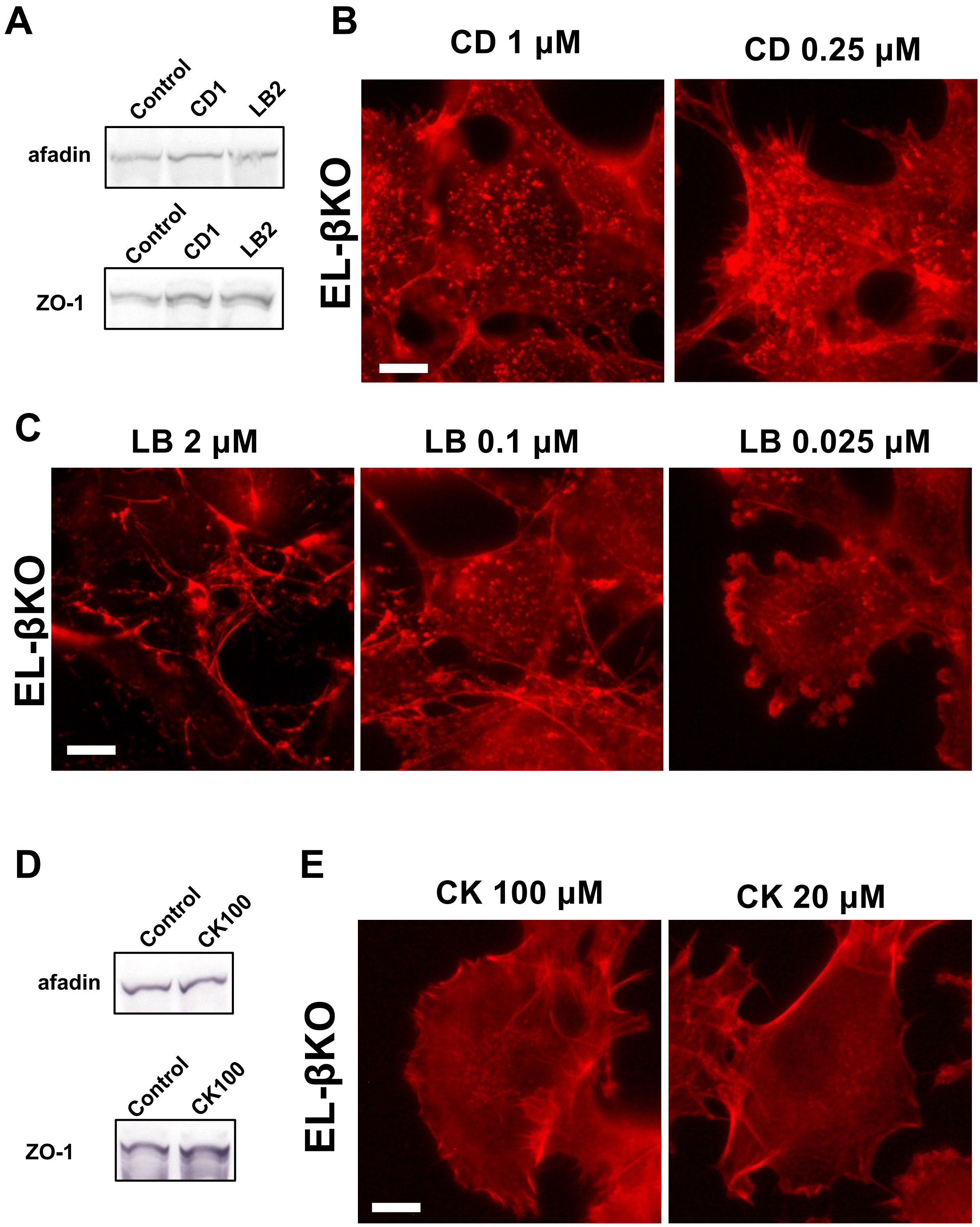

Supplementary Figure 2

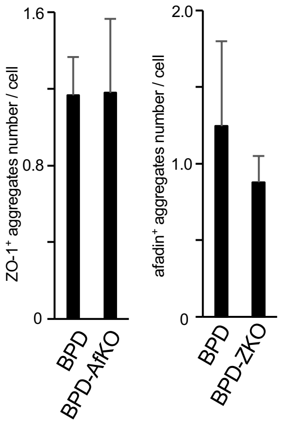

Supplementary Figure 3

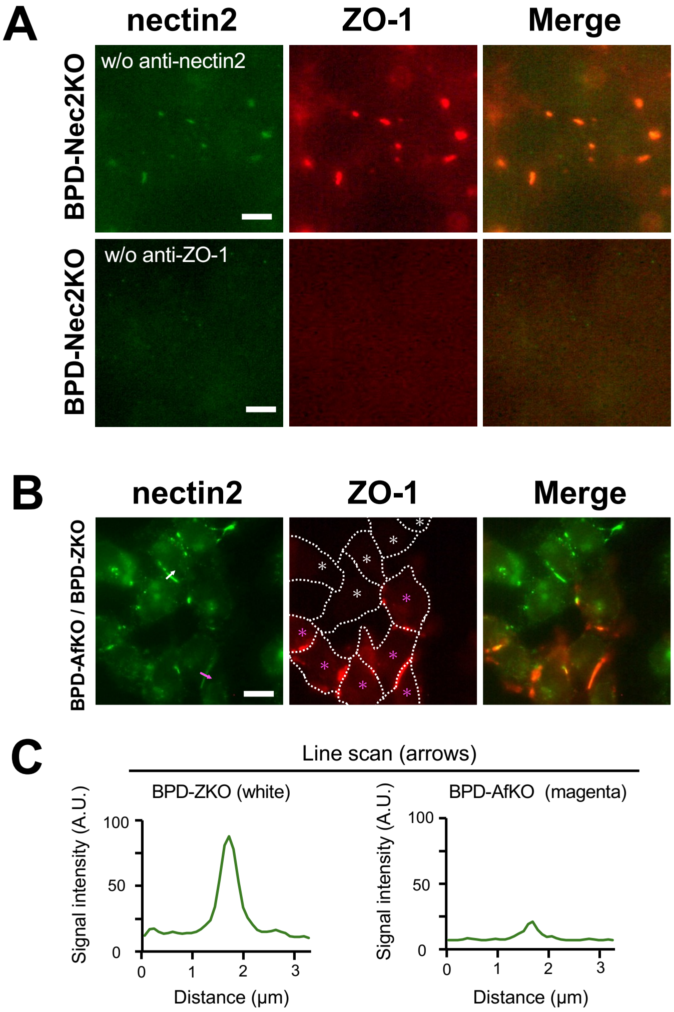

Supplementary Figure 4

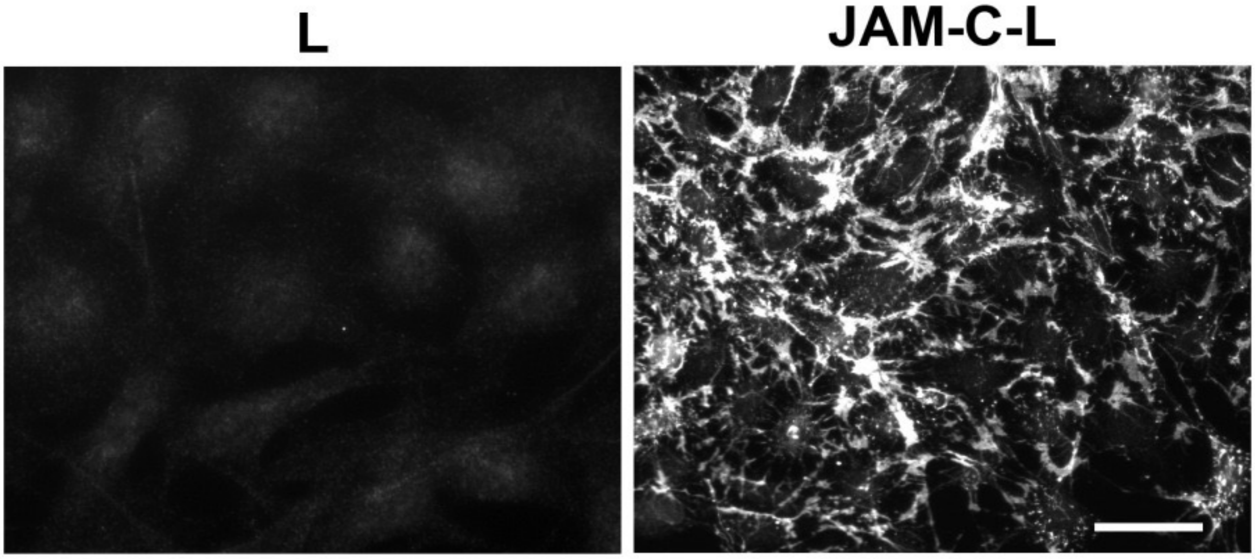

Supplementary Figure 5

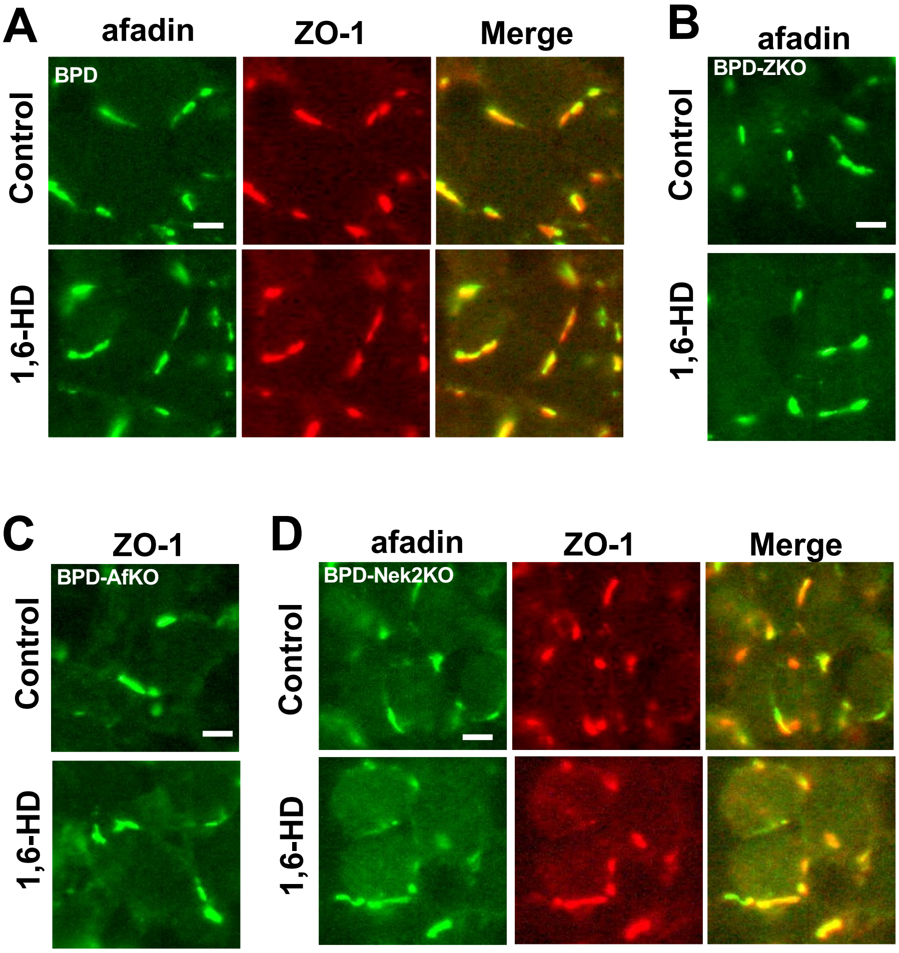

Supplementary Figure 6

Gene name	Guide sequence	Forward primer	Reverse primer
α-catenin	GAAATGACTGCCGTCCACGC	GTTGTCAAGATTGTCTCTTCATG	CTCAACCAACTCAAGTTAAGAC
β-catenin	AGCTCCTTCCCTGAGTGGCA	CAATGGGTCATTGTCGTAG	CATACTGCCCGTCAATATC
afadin	CGGGCGGCCGCGACGAAGAG	GAAAGGTCAGCTGGGATTTG	CAGGAGGCGACAAGGACGAGC
ZO-1	AATGGAGGAAACAGCTATAT	CCTTTTCTTGTGGTGTGCAGCATG	GCTACAACTAAGCACACTACCAACTGAG
ZO-2	CCTCTCAGTCATGACGACCG	GCACTATATTCACTACTGCAG	GTGCCGACTCCTCTCACTGTAG
nectin-2	CACGACGGAGCGCGTCTCTC	AGAGATGCCTGACCTGGAGA	GGGTTCCCTCCACTCACCTA

Supplementary Table 1
